# Spin-State and Reorganization Energy Considerations for Metal-Centered Photoredox Catalysis

**DOI:** 10.1021/jacs.5c14935

**Published:** 2025-10-16

**Authors:** Bekah E. Bowers, Björn Pfund, Hayden F. Beissel, Atanu Ghosh, James K. McCusker

**Affiliations:** Contribution from the Department of Chemistry, 3078 Michigan State University, 578 South Shaw Lane, East Lansing, Michigan 48824, United States

## Abstract

Transition-metal complexes featuring metal-centered excited states have recently emerged as mechanistically distinct platforms for selective photochemistry, including photoredox catalysis. Among these, Co­(III) complexes have demonstrated productive photoinduced electron transfer via the ^3^T_1_ metal-centered state. In contrast, photoreactivity from the ^5^T_2_ metal-centered state in Fe­(II) polypyridyl complexes remains limited. Building on our prior report concerning reactivity associated with the ^5^T_2_ state in [Fe­(tren­(py)_3_)]^2+^ (tren­(py)_3_ = tris­(2-pyridylmethyliminoethyl)-amine), we introduced stronger-field ligands in an effort to increase excited-state energies of Fe­(II) polypyridyl complexes and enhance reactivity. Despite achieving nanosecond-scale excited-state lifetimes and favorable thermodynamic driving forces, no photoreactivity was observed. Reinvestigation of the observations previously reported for [Fe­(tren­(py)_3_)]^2+^ revealed interactions between the metal complex and the substrate in their respective ground states that mimicked dynamic quenching of the chromophore, prompting a reassessment of mechanistic considerations inherent in leveraging reductive chemistry from the ^5^T_2_ excited state of Fe­(II). Our analysis indicates that electron transfer from the ^5^T_2_ excited state of a low-spin d^6^ metal is subject to significant barriers both in terms of reorganization energies and spin conservation that undermines its ability to act as an electron donor for photoredox catalysis. In contrast, ligand fields that are sufficient to stabilize the ^3^T_1_ excited state have available to them numerous spin-allowed and, in certain cases, near-barrierless pathways to engage in excited-state electron transfer (both oxidative and reductive depending on the identity of the metal). These results highlight the critical role of spin-state changes and their associated reorganization energy requirements in metal-centered photoredox catalysis.

## Graphical abstract

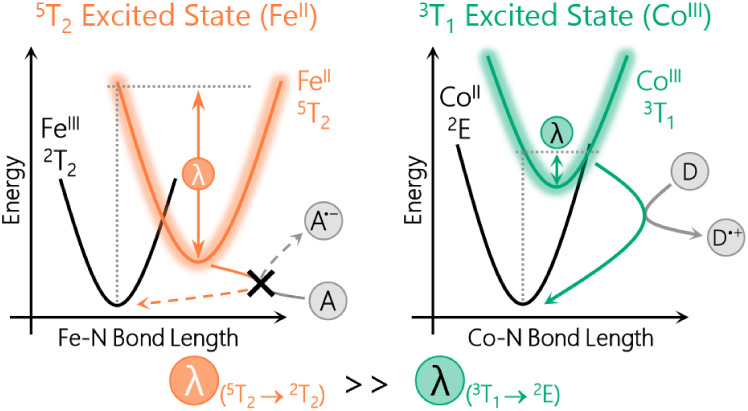

## Introduction

Photoredox catalysis has revolutionized synthetic organic chemistry by enabling energetically demanding transformations under mild conditions.^1–6^ Among the most widely used photocatalysts are Ru­(II)- and Ir­(III)-based polypyridyl complexes,^7–9^ combining tunable visible-light absorption, long-lived excited states, strong redox properties, and excellent photostability.^10,11^ These second- and third-row d^6^ polypyridyl complexes typically exhibit photoreactivity through a metal-to-ligand charge transfer (MLCT) excited state, capable of facilitating electron transfer (ET) through ligand oxidation or metal-centered reduction (Figure 1a).^12,13^ Despite their success, MLCT-based systems often lack selectivity due to the strongly reducing and oxidizing nature of the excited state. In contrast, metal-centered (MC) excited state reactivity has recently emerged as a promising strategy, offering more defined redox behavior, enabling either reductive^14–17^ or oxidative^18–21^ quenching depending on the redox properties of the metal center. In most Ru­(II)- and Ir­(III)-based complexes, MLCT excited states lie lower in energy than MC states due to strong ligand fields, often making them the lowest accessible excited states.^22–25^ In contrast, first-row transition metals typically exhibit weaker ligand fields, leading to low-lying MC states that often dominate the excited-state landscape and govern photoreactivity (Figure 1b,c).^26–30^

**Figure 1:**
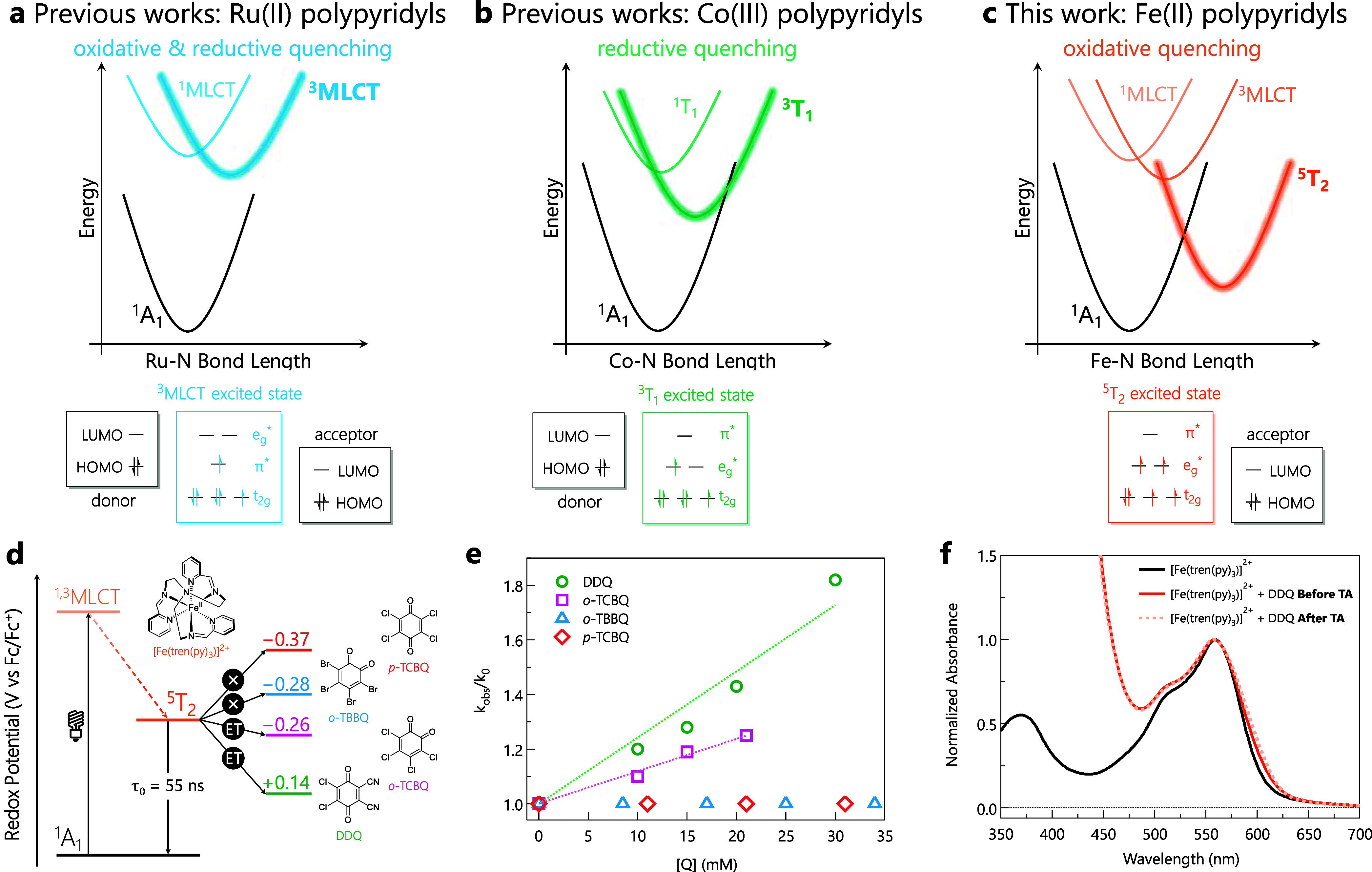
(a-c) Excited-state potential energy surface diagrams for polypyridyl complexes. (a) Ru­(II): photoactive ^3^MLCT state enables reductive electron transfer (ET) from the ligand or oxidative ET by the metal center. (b) Co­(III): photoactive metal-centered ^3^T_1_ excited state enables reductive quenching of the photosensitizer, leading to the formation of a Co­(II) species. (c) Fe­(II) excited-state cascade with its photoactive metal-centered ^5^T_2_ excited state enabling oxidative quenching of the photosensitizer, leading to formation of Fe­(III). (d) Simplified energy diagram representing previously reported reactivity from the ^5^T_2_ metal-centered excited state in [Fe­(tren­(py)_3_)]^2+^ to different benzoquinones resulting in an estimated excited-state reduction potential of −0.27 V vs Fc/Fc^+^.^18^ (e) Experimentally reproduced Stern–Volmer analysis of bimolecular reactivity between [Fe­(tren­(py)_3_)]^2+^ and benzoquinones in MeCN, using transient absorption kinetic measurements at 560 nm following photoexcitation at 580 nm. Reaction rates of 4.4 × 10^8^ M^–1^ s^–1^ and 2.2 × 10^8^ M^–1^ s^–1^ were obtained for DDQ and *o*-TCBQ, respectively, while no detectable quenching was observed for *o*-TBBQ and *p*-TCBQ. (f) Electronic absorption spectra of [Fe­(tren­(py)_3_)]^2+^ (black trace), [Fe­(tren­(py)_3_)]^2+^ with DDQ before (solid orange trace) and after (dotted orange trace) quenching experiments.

These MC states were previously considered photochemically inactive due to their low excited-state energy.^31,32^ However, recent studies from a collaborative effort between the MacMillan and McCusker groups have shown that Co­(III) polypyridyl complexes can act as potent photooxidants, activating otherwise inert substrates.^16,33^ Beyond their high excited-state reduction potentials, Co­(III) systems exhibit intrinsic photoredox selectivity in MC state reactivity, permitting only reductive quenching via the lowest energy ^3^T_1_ state (Figure 1b). Beyond Co­(III), related MC state photochemistry has been reported for Cr­(III),^15,33–39^ Mn­(IV),^40,41^ and Ni­(II)^14,42,43,44^ complexes, enabling oxidative substrate activation with notable selectivity.

In contrast, Fe­(II) polypyridyl complexes offer access to reductive substrate activation from the MC excited-state. Upon excitation into the MLCT band, these complexes undergo ultrafast decay to the MC ^5^T_2_ excited state within <200 fs (Figure 1c).^28,32^ This MC state is significantly longer-lived than the initial MLCT state, with lifetimes typically in the nanosecond range, and is proposed as the reactive state in Fe­(II) polypyridyl photocatalysis.^19,20^ However, the mechanistic basis for Fe­(II)-mediated photoredox catalysis remains unclear, given the minimal estimated Fe­(II) excited-state oxidation potentials and limited spectroscopic evidence for productive electron transfer. Elucidating this mechanism is crucial to enable rational catalyst design and to understand the origin of Fe-based photoreactivity.^45,46^

To probe the mechanistic origin of photoreactivity in Fe­(II)-polypyridyl complexes, our group examined the excited-state reactivity of [Fe­(tren­(py)_3_)]^2+^ (where tren­(py)_3_ is tris­(2-pyridyl-methyliminoethyl)­amine) with a series of benzoquinones as electron acceptors (Figure 1d).^18^ Nanosecond transient absorption spectroscopy revealed bimolecular reaction rate constants on the order of 10^8^ M^–1^ s^–1^ (Figure 1e, experimentally reproduced for this study) with an excited-state oxidation potential (*E_1/2_) of ca. −0.27 V vs Fc/Fc^+^ in MeCN. Electronic absorption spectra recorded before and after laser excitation did not reveal any changes in the optical properties of the system (Figure 1f, experimentally reproduced for this study), consistent with expectations for a photostable system undergoing reversible photoinduced ET. The zero-point energy (*E*_0,0_) of the ^5^T_2_ state was estimated using the Rehm–Weller relationship, yielding a value of approximately 0.80 eV, which is sufficient for potential applications in photoredox catalysis.^18^

In an effort to expand upon these initial studies, we sought to investigate Fe­(II) complexes with stronger-field ligands to elevate the energy of the ^5^T_2_ MC excited state and, in doing so, increase its reducing power and enhance excited-state reactivity. Although the polypyridyl complexes we employed display nanosecond excited-state lifetimes and higher zero-point energies, no photoinduced ET is observed with a range of electron acceptors despite favorable driving forces for oxidative quenching of the Fe­(II)-based ligand-field excited state. This unexpected lack of excited-state reactivity prompted a reexamination of our earlier Stern–Volmer analysis.^18^ While the Rehm–Weller relationship is well established for MLCT states and some MC states,^12,15,47,48,49^ its broader application to MC reactivity involving complex spin-state manifolds may be more subtle than previously thought.^50^ The model does not consider factors such as spin-state changes and reorganization energies, both of which appear to be especially relevant for Fe­(II) systems, leveraging reactivity from its high-spin, ^5^T_2_ ligand-field excited state, where structural and electronic rearrangements can influence excited-state dynamics.^51–55^ As we will show in this report, we believe this discrepancy arises from large reorganization energy barriers associated with spin-state changes and represents a key design consideration for photoinduced ET involving low-spin Fe­(II) polypyridyl complexes.

## Results

### Tuning Fe­(II) Polypyridyl-Based Photoreactivity

Building on our previous report of photoinduced ET transfer from the ^5^T_2_ state in [Fe­(tren­(py)_3_)]^2+^ (Figure 1d–f),^18^ we investigated a series of Fe­(II) complexes featuring stronger-field polypyridyl ligands with the goal of enhancing the observed reactivity (Figure 2a). We began with [Fe­(bpy)_3_]^2+^ (bpy = 2,2’-bipyridine), a low-spin Fe­(II) complex that presents a stronger ligand field than [Fe­(tren­(py)_3_)]^2+^ due to the replacement of the three imine donors with pyridine-based ligands. Based on the ground-state redox potential of [Fe­(bpy)_3_]^2+^ (*E*_1/2_ = 0.68 V vs Fc/Fc^+^) and an estimated zero-point energy of *E*_0,0_ = 0.94 eV,^31^ the excited-state redox potential (*E_1/2_) is calculated to be approximately −0.26 V vs Fc/Fc^+^ (Figure 2b), which is similar to that predicted for [Fe­(tren­(py)_3_)]^2+^ (*E_1/2_ = −0.27 V vs Fc/Fc^+^).^18^ Therefore, it is predicted to reduce DDQ (*E*_1/2_ = 0.14 V vs Fc/Fc^+^)^18,56^ with a thermodynamic driving force (Δ*G* ^0^_ET_) of approximately −0.4 eV. The comparable driving forces for oxidative quenching of [Fe­(bpy)_3_]^2+^ and [Fe­(tren­(py)_3_)]^2+^ stems from changes in the ground-state redox properties between the two compounds, despite the differences in ligand-field strengths.

**Figure 2:**
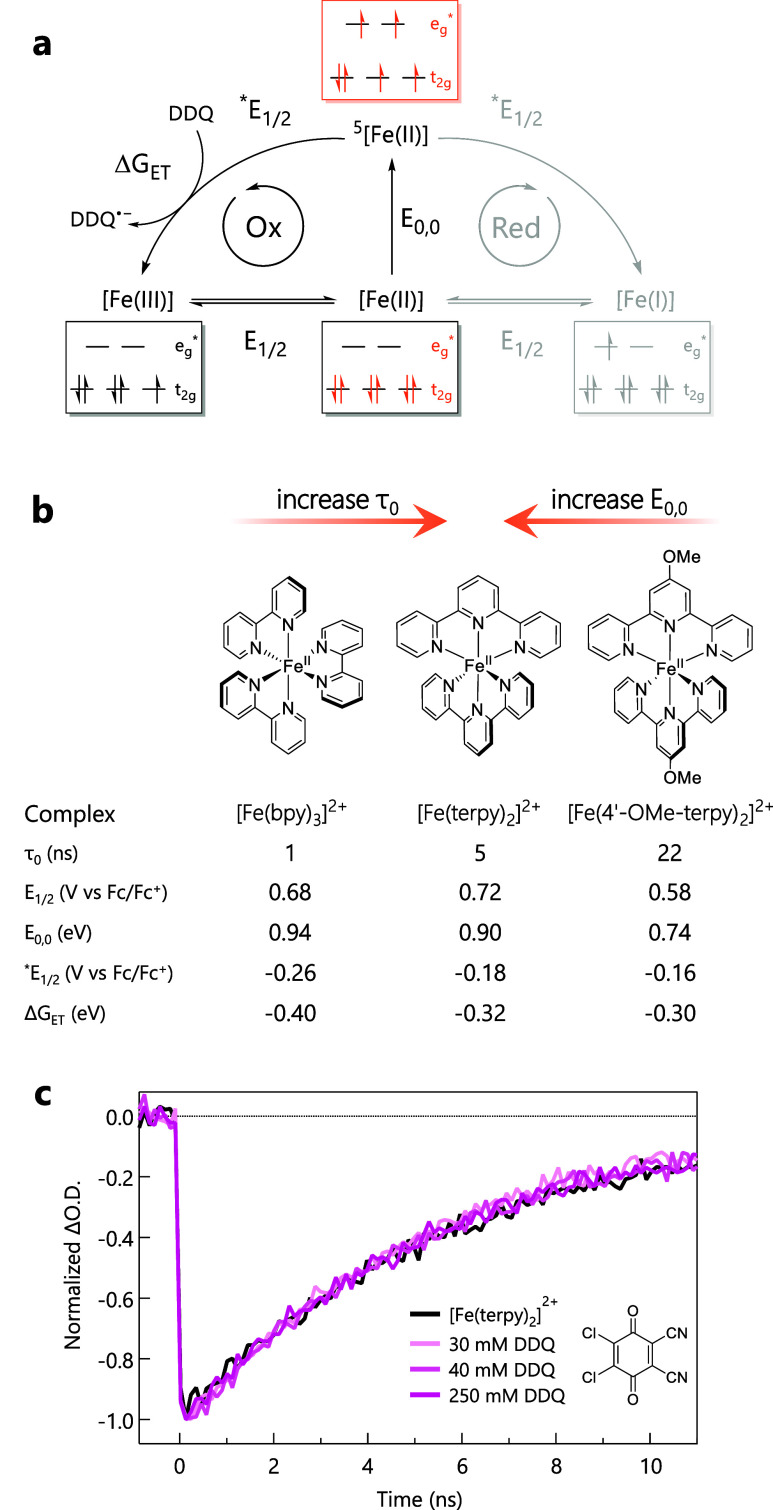
(a) Latimer diagram for Fe­(II) polypyridyl chromophores with ^5^T_2_ lowest excited state with *E*_0,0_ representing the zero-point energy, and *E*_1/2_ and *E_1/2_ the ground and excited-state redox potentials, respectively. The oxidative cycle is shown in black, whereas the reductive cycle is represented on the right in gray. The relevant electron configurations are indicated, assuming O_h_ symmetry. (b) Fe­(II) polypyridine complexes with the corresponding excited-state lifetime (τ_0_), ground-state redox potential (*E*_1/2_), zero-point energy (*E*_0,0_), excited-state oxidation potential (*E_1/2_), and the corresponding driving force (Δ*G*_ET_) for reduction of DDQ. All relevant data were obtained in MeCN at 20 °C and can be found in Supporting Information. (c) Transient absorption kinetics at 560 nm for deaerated MeCN solutions containing [Fe­(terpy)_3_]^2+^ and varying concentrations of DDQ (0 to 250 mM), following excitation at 530 nm.

Time-resolved absorption kinetics were recorded at 480 nm following excitation at 520 nm of [Fe­(bpy)_3_]^2+^ in MeCN solutions in both the absence and presence of 30 mM DDQ. The observed kinetics were found to be unaffected by the presence of DDQ (Figure S1), indicating the absence of efficient photoinduced ET (or any reactivity, for that matter) under these conditions. This was surprising, as the driving force of 0.4 eV is expected to yield a diffusion-limited bimolecular quenching rate constant of ∼1.9 × 10^10^ M^–1^ s^–1^;^57^ even given the substantially reduced excited-state lifetime of the ^5^T_2_ state of [Fe­(bpy)_3_]^2+^ (i.e., ∼1 ns versus ∼55 ns for [Fe­(tren­(py)_3_)]^2+^), this should still lead to a ∼35% reduction in the observed time constant due to dynamic quenching of the ligand-field excited state. However, if the actual quenching rate constant were closer to 4.4 × 10^8^ M^–1^ s^–1^, i.e., similar to that previously reported for [Fe­(tren­(py)_3_)]^2+^ with DDQ,^18^ only ∼1% quenching would be expected under these conditions: such a small change in lifetime would likely elude detection. Unfortunately, increasing the DDQ concentration to compensate for the shorter lifetime of [Fe­(bpy)_3_]^2+^ and enhance the yield was not feasible due to the strongly absorbing nature of DDQ and the corresponding interference with selective excitation of [Fe­(bpy)_3_]^2+^ (Figure S1).

We therefore turned to [Fe­(terpy)_2_]^2+^ (terpy = 2,2’:6’,2″-terpyridine), which retains a ligand-field strength comparable to that of [Fe­(bpy)_3_]^2+^ but features a longer ^5^T_2_ excited-state lifetime (5 ns).^31^ Additionally, its MLCT absorption envelope is significantly red-shifted as compared to [Fe­(bpy)_3_]^2+^, thereby allowing selective excitation of the chromophore in the presence of higher concentrations of DDQ (Figure S2). Based on the known ground-state potential (*E*_1/2_= 0.72 V vs Fc/Fc^+^) and the zero-point energy (*E*_0,0_ = 0.90 eV)^31^ the excited-state oxidation potential is estimated to be −0.18 V vs Fc/Fc^+^, corresponding to a driving force of ca. −0.32 eV for photoinduced ET to DDQ. Upon 560 nm photoexcitation, transient absorption kinetics at 530 nm were recorded for [Fe­(terpy)_2_]^2+^ in MeCN solutions that range as high as 250 mM in DDQ. However, no changes in the kinetics were observed (Figure 2c). Based on a bimolecular quenching rate constant of 4.4 × 10^8^ M^–1^ s^–1^ (again, similar to that observed for [Fe­(tren­(py)_3_)]^2+^)^18^ and the 5 ns intrinsic lifetime for the ^5^T_2_ excited state, these conditions should result in a ∼35% reduction in the measured lifetime.^58^ A change in lifetime from 5 ns to ∼3.25 ns is well within our experimental capabilities to detect; therefore, the absence of detectable quenching indicates inefficient (or nonexistent) ET despite conditions that should be amenable for the observation of oxidative quenching of the excited ligand-field state of [Fe­(terpy)_2_]^2+^.

As a final effort to understand this increasingly confusing set of results, we carried out analogous studies using [Fe­(4’-OMe-terpy)_2_]^2+^ (4’-OMe-terpy = 4’-methoxy-2,2’:6’2″-terpyridine) as the chromophore. This compound, which is structurally homologous to [Fe­(terpy)_2_]^2+^, is characterized by a significantly longer-lived ^5^T_2_ excited state (22 ns as compared to 5 ns for the latter) due to π-donation from the -OMe group and a concomitant decrease in the ligand-field strength associated with the ligand.^59,60^ Unlike [Fe­(bpy)_3_]^2+^ or [Fe­(terpy)_2_]^2+^, the excited-state potential of [Fe­(4’-OMe-terpy)_2_]^2+^ could not be directly obtained from literature since an *E*_0,0_ value for the ^5^T_2_ state has, to our knowledge, not been published. We therefore employed semiclassical Marcus theory to estimate *E*_0,0_;^31,61^ since the difference between 4’-OMe-terpy and terpy lies only in the secondary coordination sphere,^31,61^ we considered it reasonable that the electronic coupling constant and reorganization energy of 6 ± 1 cm^–1^ and 14 000 ± 1000 cm^–1^, respectively, that we reported^31^ for [Fe­(terpy)_2_]^2+^ can be assumed for [Fe­(4’-OMe-terpy)_2_]^2+^. This analysis yielded an estimated *E*_0,0_ value of 0.74 eV for [Fe­(4’-OMe-terpy)_2_]^2+^, approximately 0.2 eV lower than that of [Fe­(terpy)_2_]^2+^ (Figure S3). Applying the Rehm–Weller relationship with a ground-state *E*_1/2_ of 0.58 V vs Fc/Fc^+^, the driving force for ET from the excited state of [Fe­(4’-OMe-terpy)_2_]^2+^ to DDQ was calculated to be −0.30 eV: we believe this should be sufficient to observe excited-state quenching.

Selective excitation at 570 nm and monitoring the transient absorption kinetics at 550 nm again revealed no changes in the lifetime of the Fe-based excited state at DDQ concentrations up to 250 mM (Figure S3). Assuming that any quenching resulting in less than ∼5% reduction of the excited-state lifetime would likely escape detection,^62^ this indicates that the bimolecular ET rate constant must be slower than ∼10^7^ M^–1^ s^–1^. Such a slow ET rate would seem to be inconsistent with the favorable thermodynamic driving force that exists for the proposed reaction, especially given the (relative) accessibility of the metal center in terpy-based complexes.

### Revisiting ^5^T_2_ Excited-State Reactivity of [Fe­(tren­(py)_3_)]^2+^

The unexpected lack of excited-state reactivity across the Fe­(II) polypyridyl series just described prompted us to revisit our previous study involving what we reported to be electron transfer between the excited state of [Fe­(tren­(py)_3_)]^2+^ and benzoquinones.^18^ Photoinduced dynamics subsequent to MLCT excitation of [Fe­(tren­(py)_3_)]^2+^ was assessed using nanosecond time-resolved electronic absorption spectroscopy by comparing the observed lifetime of the ^5^T_2_ ligand-field excited state—which is known to be the lowest energy excited state for this compound and is formed in <200 fs^28,32,63,64^ —in the presence and absence of substituted benzoquinones spanning a range of reduction potentials (Figure 1d). A Stern–Volmer analysis indicated bimolecular quenching behavior (Figure 1e), yielding results comparable to those previously reported.^18^ Steady-state electronic absorption spectra of the reaction mixtures were recorded before and after the transient absorption measurements (Figure 1f). No spectral changes were observed, an observation that we interpreted to mean that no net photochemistry was occurring in these systems (i.e., the overall process ultimately led to the reformation of the starting materials, namely [Fe­(tren­(py)_3_)]^2+^ and the starting benzoquinone, due to back-electron transfer). Similar to our previous study, the Stern–Volmer plots exhibited relatively large experimental uncertainty; multiple runs were carried out in order to improve confidence limits, but the reported results reflect the significant standard deviations that characterized these measurements. A drawback of the previous study—which was discussed in that earlier report—was our inability to detect photoproducts such as [Fe­(tren­(py)_3_)]^3+^ or the semiquinone forms of the quenchers. We believed this was due to spectral overlap among the absorption cross sections of the ground states of [Fe­(tren­(py)_3_)]^2+^ and the various benzoquinones, and the redox pairs resulting from photoinduced electron transfer (the latter having been established through spectroelectrochemical measurements). The mechanistic assignment of excited-state electron transfer was therefore based on (1) the well-behaved kinetics consistent with dynamic quenching of the ^5^T_2_ ligand-field excited state of [Fe­(tren­(py)_3_)]^2+^, (2) the absence of net photochemistry, suggesting there was no photoinduced decomposition, (3) the spin-forbidden and endothermic nature of any energy transfer pathway involving the ^5^T_2_ excited state and low-energy triplet excited state(s) of the benzoquinones, and (4) the experimental correlation between the measured quenching rate constants and the redox potentials of the benzoquinones that could be well-described by classical Marcus Theory. Nevertheless, the fact that the series of Fe­(II) polypyridyls described in the previous sections showed *no evidence of photoinduced reactivity of any kind* despite possessing properties similar to (and in some cases more favorable than) that of [Fe­(tren­(py)_3_)]^2+^ compelled us to examine the chemistry of the [Fe­(tren­(py)_3_)]^2+^/benzoquinone system in greater detail.

Given the invariance in the electronic absorption spectrum before and after the Stern–Volmer quenching studies and the fact that the oxidation potential of Fe­(II) in [Fe­(tren­(py)_3_)]^2+^ and the reduction potential of DDQ made electron transfer involving the ground states of these compounds significantly endothermic, our previous study did not include a detailed investigation of any reactivity of the donor/acceptor mixtures prior to photoexcitation. As part of the current reassessment, we acquired ^1^H NMR spectra of a solution of [Fe­(tren­(py)_3_)]^2+^ containing an excess of DDQ in *d*_3_-MeCN where the solution was kept in the dark at room temperature. Within approximately 5 min of preparing the solution, new spectral features appeared in both the aromatic and aliphatic regions that increased steadily over time (Figure 3b). This is a clear indication that [Fe­(tren­(py)_3_)]^2+^ does, in fact, react with DDQ in the absence of light, an observation that significantly undermines our confidence in the conclusions drawn from our previous study. To probe whether this behavior is specific to DDQ, analogous ^1^H NMR experiments were performed with *o*-tetrachlorobenzoquinone (*o*-TCBQ), a quencher previously shown to reduce the excited-state lifetime of [Fe­(tren­(py)_3_)]^2+^ (Figure 1e).^18^ New ^1^H NMR signals identical to those observed following the addition of DDQ were detected (Figures S4 and S5), suggesting that ground-state reactivity is not limited to DDQ. Additional ^1^H NMR experiments were conducted in the presence of *o*-tetrabromobenzoquinone (*o*-TBBQ) and *p*-tetrachlorobenzoquinone (*p*-TCBQ). These two substrates did not affect the excited-state lifetime of [Fe­(tren­(py)_3_)]^2+^ (Figure 1e).^18^ We found that *o*-TBBQ exhibits reactivity analogous to both DDQ and *o*-TCBQ but at a significantly slower inferred rate based on the amount of time that is needed to elapse before the reaction products became evident in the NMR spectrum (Figure S6). No change in the ^1^H NMR spectrum of mixtures of [Fe­(tren­(py)_3_)]^2+^ and *p*-TCBQ were observed (Figure S7), indicating that whatever reaction was occurring with the other three benzoquinones was not occurring in this case of this substrate. The trend in ground-state reactivity just described qualitatively matches that found in the Stern–Volmer experiments, i.e., fastest with DDQ to no reaction with *p*-TCBQ. These observations suggest that the change in the excited-state lifetime of [Fe­(tren­(py)_3_)]^2+^ codified in Figure 1e is likely related to ground-state reactivity rather than photoinduced ET from the ^5^T_2_ excited state of [Fe­(tren­(py)_3_)]^2+^.

**Figure 3:**
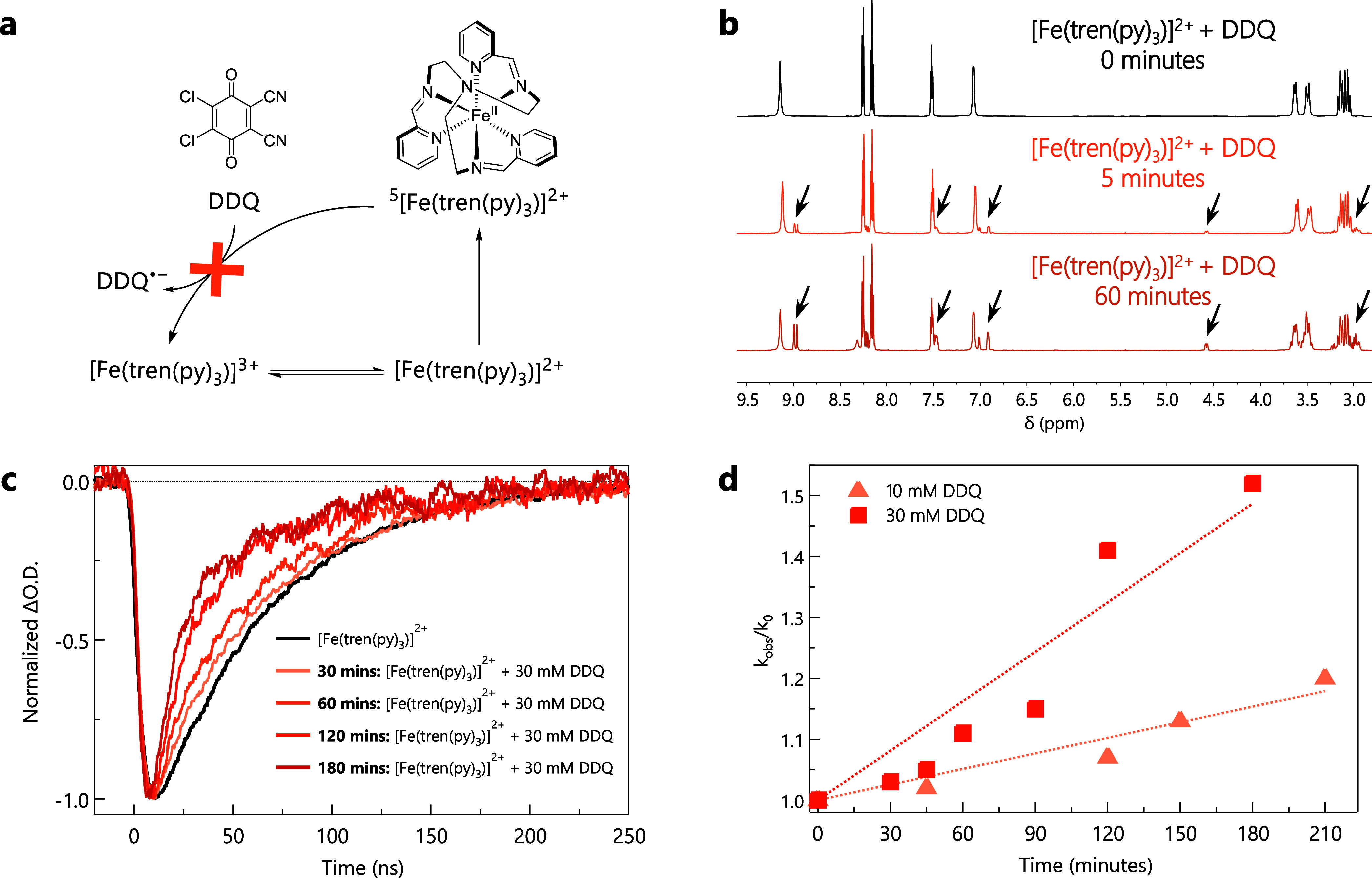
(a) Latimer diagram for [Fe­(tren­(py)_3_)]^2+^ in MeCN. Redox potentials given in V vs Fc/Fc^+^. (b) ^1^H NMR spectra recorded at different time points for a *d*_3_-MeCN solution containing [Fe­(tren­(py)_3_)]^2+^ and DDQ, showing a continuous change in composition over time. Arrows indicate new peaks associated with the formation of new products. (c) Transient absorption kinetics at 560 nm following 580 nm excitation, at different time points (0, 30, 60, 120, 180 min) on a sample containing 0.05 mM [Fe­(tren­(py)_3_)]^2+^, 30 mM DDQ, and 0.1 M TBAPF_6_ in deaerated MeCN. (d) Stern–Volmer-type analysis based on the kinetic traces of the type shown in (c), using time on the *x*-axis instead of quencher concentration. Data for solutions containing [Fe­(tren­(py)_3_)]^2+^ and DDQ at concentrations of 10 mM (triangles) and 30 mM (squares) are illustrated.

Correlation is not causation, so to evaluate whether the ground-state reactivity is directly linked to the observed lifetime decrease of [Fe­(tren­(py)_3_)]^2+^, transient absorption kinetics were recorded as a function of mixing time at constant DDQ concentration (Figure 3c). After 30 min, the lifetime of [Fe­(tren­(py)_3_)]^2+^ decreased from 55 ns to 53 ns and continued to decrease to 35 ns after 180 min. A 3-fold reduction in the DDQ concentration leads to a slower apparent rate in lifetime attenuation (i.e., it takes longer to observe a comparable lifetime reduction for solutions containing 10 mM DDQ as compared to 30 mM), consistent with expectations for a simple, thermally driven bimolecular reaction between [Fe­(tren­(py)_3_)]^2+^ and the benzoquinone. This was quantified through what we describe as a pseudo-Stern-Volmer analysis by plotting the ratio of the natural excited-state lifetime of [Fe­(tren­(py)_3_)]^2+^ to its lifetime in the presence of DDQ at different mixing times (Figure 3d). The similarity between this plot and Figure 1e provides compelling evidence that the previously reported quenching behavior is most likely the result of ground-state reactivity between [Fe­(tren­(py)_3_)]^2+^ and DDQ, rather than from photoinduced ET associated with the ^5^T_2_ excited ligand-field state of the chromophore.

As mentioned previously, the UV–vis absorption spectra associated with the MLCT band of the [Fe­(tren­(py)_3_)]^2+^ between 500 and 650 nm remained largely unchanged before and after transient absorption measurements (Figure 1f). A similar persistence of the optical properties of the Fe­(II)-based chromophore following the time-dependent NMR mixing experiments described above was also observed. It is important to note that the aforementioned NMR spectra were all characterized by sharp, well-resolved resonances with no indication of the presence of a paramagnetic species. Moreover, changes in the NMR spectra eventually stopped and remained largely invariant for at least 24 h, suggesting that some moderately stable species is ultimately formed (Figure S8). The MLCT absorption that dominates the visible spectrum of [Fe­(tren­(py)_3_)]^2+^ corresponds to a (t_2g_)^6^(π*)^0^ → (t_2g_)^5^(π*)^1^ transition involving the π* orbital(s) associated with the pyridine rings of the ligand.^63^ It is red-shifted relative to a compound such as [Fe­(py)_6_]^2+^ (py = pyridine)^65^ due in part to conjugation between the pyridine rings and their adjacent imine groups in the tren­(py)_3_ ligand, as well as ligand-field-induced destabilization of the Fe­(II) t_2g_ orbital caused by the imine binding motif. The fact that this absorption feature does not appear to be affected over the course of these studies suggests that whatever reaction is occurring between [Fe­(tren­(py)_3_)]^2+^ and the benzoquinones, it appears to leave the primary coordination sphere of the metal complex largely intact.

This apparent lack of involvement of the pyridine-imine fragments or the metal center itself leaves the tertiary amine of the tren­(py)_3_ ligand as the most likely site of reactivity. While not the case for Fe­(II)-based complexes of this ligand, tren­(py)_3_ yields a 7-coordinate complex when bound to Zn­(II),^66,67^ indicating that the lone pair on the amine has the potential to be stereochemically active. We therefore carried out a simple experiment of mixing Me_6_-tren (Me_6_-tren = tris­[2-dimethylamino)­ethyl]­amine) with DDQ; the hexamethyl version was used to suppress any potential reaction between the primary amines of tren and the benzoquinone. Upon mixing, the solution immediately turned dark purple with a ^1^H NMR spectrum that reveals changes in the resonances associated with Me_6_-tren (Figure S9). The spectral features are sharp, suggesting the formation of a charge-transfer adduct between the two molecules as opposed to an electron transfer reaction, which would produce two paramagnetic species (i.e., a tertiary amine radical and a semiquinone). We suspect a similar process is likely occurring between the tertiary amine of [Fe­(tren­(py)_3_)]^2+^ and the benzoquinones; the apparent correlation with the redox potential of the benzoquinone would then reflect an attenuation in the strength of the donor–acceptor interaction as the reduction potential of the benzoquinone becomes more negative. We note that such an interaction would likely have little influence on the MLCT absorption characteristics of the compound, consistent with the observations described above. Unfortunately, we have been unsuccessful in our efforts to isolate the new species being formed and are therefore unable to provide an exact formulation for this compound.

While the decrease in excited-state lifetime clearly correlates with the accumulation of the [Fe­(tren­(py)_3_)]^2+^/benzoquinone ground-state adduct, the mechanism responsible for the reduction in lifetime remains unclear. One possibility is that the interaction could quench the ^5^T_2_ excited state of free, photoexcited [Fe­(tren­(py)_3_)]^2+^ in what could be characterized as dynamic quenching via self-exchange. However, we believe this is doubtful given the relatively low concentration of [Fe­(tren­(py)_3_)]^2+^ (∼50 μM), which is far below the millimolar concentrations typically required to observe measurable diffusion-controlled quenching.^58,68,69^ More likely, we believe that adduct that is being formed between [Fe­(tren­(py)_3_)]^2+^ and the benzoquinone itself is being photoexcited, a reasonable expectation given that the MLCT absorption envelopes of the adduct and the parent Fe­(II) complex are essentially identical. If the metal-based ^5^T_2_ excited state of the Fe­(II)/benzoquinone adduct possesses a different lifetime than free [Fe­(tren­(py)_3_)]^2+^, the time-resolved absorption data would display biphasic kinetics subject to (1) the difference in the two compounds’ intrinsic lifetimes and whether the observed kinetics can reasonably support inclusion of a second component, and (2) the relative concentrations of the two chromophores, the latter being reflected in the ratio of the pre-exponential factors from a fit to a biphasic kinetic model assuming such a model is justified. Given these considerations, we believe that the most straightforward explanation for our observations is that the [Fe­(tren­(py)_3_)]^2+^/benzoquinone ground-state adduct has a shorter excited-state lifetime than free [Fe­(tren­(py)_3_)]^2+^. When the adduct is present, but at sufficiently low concentration, the data can be reasonably fit to a single exponential kinetic model. As the amount of adduct present increases but remains too low to clearly manifest as a second component in the data, the fitted lifetime will begin to decrease, reflecting a weighted average of the lifetimes of both species (Figure S10). Finally, as the adduct becomes more concentrated at extended mixing times, the data can no longer be fit to a single exponential as a second component emerges. This becomes more evident when the data is fit logarithmically, where the deviation from linearity is evidence of two components (Figure S11). This scenario is also realized in Figure 3c, data which we believe supports this interpretation.

While we cannot specify the exact nature of the species being formed upon combining [Fe­(tren­(py)_3_)]^2+^ and various substituted benzoquinones, the results just described clearly indicate that our previous interpretation of oxidative quenching from the ^5^T_2_ excited ligand-field state of [Fe­(tren­(py)_3_)]^2+^ was incorrect. Instead, the observed lifetime decrease arises from the formation of an adduct between [Fe­(tren­(py)_3_)]^2+^ and the benzoquinones used in the Stern–Volmer studies that possess an MLCT absorption band identical to that of the primary chromophore but is characterized by a ^5^T_2_ ligand-field excited state with a shorter lifetime. Buildup of this latter species over the course of the experiment gave the appearance of dynamic quenching by the substrate, which ultimately led to a misinterpretation of the results.

## Discussion

The determination that the lowest energy excited state of [Fe­(tren­(py)_3_)]^2+^ does *not* engage in excited-state electron transfer immediately raised what we view as a far more substantive issue: why, despite nanosecond time-scale lifetimes for their ^5^T_2_ excited ligand-field states and thermodynamically favorable redox potentials for oxidative quenching by DDQ, is there no photoreactivity observed for *any* of the Fe­(II) polypyridyl complexes we have investigated? This lack of reactivity suggests that there may be factors involved in electron transfer reactions leveraging certain types of metal-centered ligand-field excited states such that the Rehm–Weller formalism may not reliably predict ET thermodynamics.^17^ This would stand in stark contrast to its broad applicability when applied to MLCT excited states and would represent an important design consideration for chromophores proposed to leverage excited states of this nature. To explore this hypothesis, we compared potential electron transfer from the ^5^T_2_ excited state endemic to Fe­(II) polypyridyl complexes with their valence isoelectronic Co­(III) counterparts, where reactivity stemming from their ^3^T_1_ ligand-field excited states is well established.^16,33,70^

### Photoinduced Electron Transfer from the ^3^T_1_ Excited State in Co­(III) Complexes

We focus first on the previously reported complex, [Co­(Br_2_-bpy)_3_]^3+^ (Br_2_-bpy = 4,4’-dibromo-2,2’-bipyridine), where reductive quenching of a MC ^3^T_1_ excited state has been well established. Bimolecular quenching studies incorporating this compound as a sensitizer were used to determine an excited-state reduction potential in the range of +1.2 to +1.3 vs Fc/Fc^+^.^16^ Considering the ground-state reduction potential of −0.1 V vs Fc/Fc^+^,^16,33^ the Rehm–Weller formalism predicts an estimated *E*_0,0_ of approximately 1.3 eV, in good agreement with DFT calculations.^61^

Due to the lack of observed emission from the ^3^T_1_ excited state of [Co­(Br_2_-bpy)_3_]^3+^, the value for *E*_0,0_ quoted above cannot be directly verified in a manner analogous to what one would do for an emissive charge-transfer complex like [Ru­(bpy)_3_]^2+^. To obtain experimental confirmation, we turned instead to data on [Co­(MeImP)_2_]^+^ (MeImP = 1,1′-(1,3-phenylene)­bis­(3-methyl-1-imidazole-2-ylidene)), which exhibits a well-defined MC-based emission band at 77 K with an experimentally determined *E*_0,0_ of 1.90 eV;^71^ the significantly higher energy reflects the stronger σ-donating character of the imidazolium ligands in [Co­(MeImP)_2_]^+^, resulting in a substantially enhanced ligand field strength.^72–75^ If we make the assumption that the multielectronic term states stemming from the same one-electron configuration track parallel to each other with changes in ligand-field strength—an assumption supported by the fact that the slopes of these term states are identical in the d^6^ Tanabe-Sugano diagram—we can infer the energy of the ^3^T_1_ state in [Co­(Br_2_-bpy)_3_]^3+^ by using the emission data on [Co­(MeImP)_2_]^+^ and the electronic absorption spectra of both compounds. The lowest energy spin-allowed ligand-field band of [Co­(MeImP)_2_]^+^, namely the ^1^A_1_ → ^1^T_1_ absorption, is ∼6000 cm^–1^ (∼0.74 eV) higher in energy than the corresponding transition in [Co­(Br_2_-bpy)_3_]^3+^ (Figure S12). Subtracting this energy difference from the experimentally observed *E*_0,0_ value of [Co­(MeImP)_2_]^+^ (1.90 eV) yields an *E*_0,0_ of approximately 1.2 eV for the ^3^T_1_ state of [Co­(Br_2_-bpy)_3_]^3+^, which is in good agreement with the value derived above from the Rehm–Weller analysis.^16^ We suggest that this agreement, together with previously reported DFT calculations,^61^ provides indirect experimental support for the estimated *E*_0,0_ of [Co­(Br_2_-bpy)_3_]^3+^ and validates the use of the Rehm–Weller relationship for assessing the energetics of the photoredox-active ^3^T_1_ states in Co­(III) polypyridyl complexes.

Ligand-field excited states are characterized by a reorganization of electrons among the d-orbitals of the metal center (as opposed to the kind of charge separation one normally considers when envisioning photoinduced chemical potential for an electron transfer reaction). An important consequence of this is that there exist multiple pathways involving changes in both spin state and equilibrium geometry stemming from the redistribution of electrons among π-bonding/antibonding and σ-antibonding orbitals that must be considered. As the following discussion will reveal, these considerations provide considerable insight into both the observed reactivity of certain classes of ligand-field excited states as well as the unexpected absence of reactivity for others that, based on a classic Rehm–Weller analysis, should engage in exothermic electron transfer.

We first consider the case of Co­(III) polypyridyl complexes, which have been shown to undergo photoinduced electron transfer in the form of reductive quenching of its ^3^T_1_ ligand-field excited state. Figure 4 depicts the two most likely pathways by which this excited-state electron transfer event can proceed.^76,77^ Upon formation of a contact pair and reduction of the ^3^T_1_ excited state, the electronic configuration of the resulting Co­(II) species can be represented as either (t_2g_)^5^(e_g_*)^2^ (*S* = ^3^/_2_, path 1, green box) or (t_2g_)^6^(e_g_*)^1^ (*S* = ^1^/_2_, path 2, yellow box). Exchange interactions between Co­(II) and the substrate radical that will be created immediately following electron transfer, but prior to cage escape, will give rise to two electronic states in each case (*S*_T_ = 1 and 2 for path 1 and *S*_T_ = 0 and 1 for path 2);^78^ a reaction trajectory that conserves spin would presumably proceed through the *S*_T_ = 1 states in both scenarios. Since [Co­(bpy’)_3_]^2+^ complexes are high spin,^79–81^ cage escape along path 1 directly yields the experimentally observed products. In contrast, path 2 requires conversion from the initially formed low-spin species to the high-spin form of Co­(II) at some point in the course of the reaction (Figure 4, path 2b). Vura-Weis and coworkers have studied an analogous spin-state conversion process in cobalt-containing cubanes using M-edge time-resolved X-ray spectroscopy and found it to occur on a subpicosecond time scale.^82^ We therefore suggest that the low-spin to high-spin conversion will happen prior to cage escape, meaning that path 2 will ultimately converge with path 1 to yield the observed photoproducts. The inner-sphere reorganization energy for the overall process (λ_i_) will be dominated by the addition of a second electron in the e_g_* σ-antibonding orbitals, which happens along each of the two trajectories, albeit at different stages (Figure 4, path 1a and 2b). In an absolute sense, the addition of an additional step in path 2 prior to the spin flip should give rise to a slightly larger value for λ_i_ arising from the addition of an electron to the t_2g_ orbitals but given the generally weak nature of π-interactions between polypyridyl ligands and first-row metal complexes^83–85^ we expect this difference to be relatively small. More significant is the fact that path 2 involves the initial formation of what would be an excited electronic state of the Co­(II) polypyridyl complex, which would presumably make path 1 a more thermodynamically favorable trajectory.

**Figure 4:**
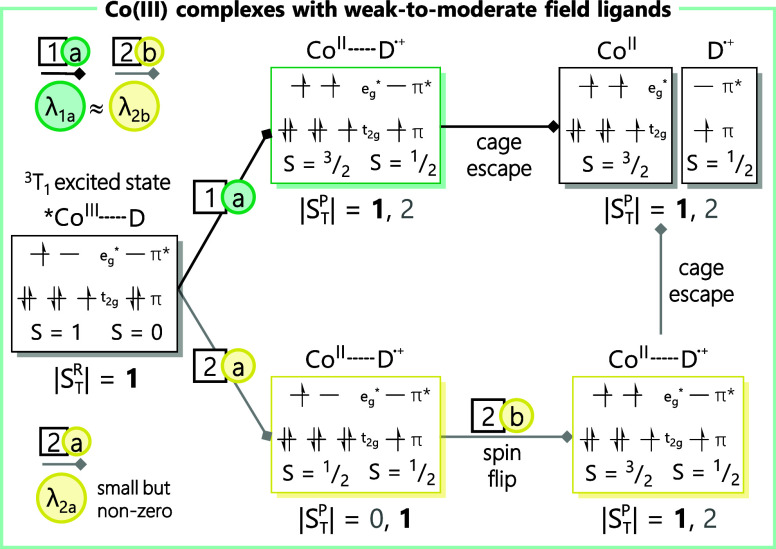
Simplified consideration of spin selection rules for photoinduced electron transfer from a donor (D) to the ^3^T_1_ excited state of Co­(III) complexes containing weak-to-moderate field ligands. Total spin of the reactant (| $S_{T}^{R}$ |) and the product (| $S_{T}^{P}$ |) was determined by considering the individual microspin states (S) for the cobalt species and donor based on a formalism described in reference 80.

### Photoinduced Electron Transfer from the ^5^T_2_ Excited State in Fe­(II) Complexes

With the formalism developed in the preceding section in place, we now turn our attention to the case of oxidative quenching of the ^5^T_2_ excited state of Fe­(II) polypyridyls. The overriding issue underpinning photoredox involving this class of compounds revolves around reorganization energy. Compounds typically invoked for use in photoredox catalysis possess reasonably strong absorption features, particularly in the visible region of the spectrum. This condition is satisfied with low-spin Fe­(II) polypyridyl complexes; high-spin Fe­(II) compounds typically exhibit charge-transfer absorptivities that are an order of magnitude smaller than their low-spin counterparts due to the longer metal–ligand bond distance for high-spin Fe­(II) complexes (ca. 0.2 Å) and the concomitant reduction in metal–ligand orbital overlap.^51,86^ Accordingly, low-spin Fe­(II) complexes are the preferred option as potential chromophores for photoredox applications. Electron transfer involving either the ground or excited state(s) of an Fe­(II) complex will be preferentially oxidized due to the prohibitively negative reduction potential associated with the formation of what would formally be an Fe­(I) species. Because the ground state of the compound prior to photoexcitation is low-spin (i.e., a ^1^A_1_ state stemming from a (t_2g_)^6^(e_g_*)^0^ configuration), the ground state of the Fe­(III) complex formed subsequent to electron transfer will invariably be low-spin as well (i.e., a ^2^T_2_ state derived from (t_2g_)^5^(e_g_*)^0^).

Oxidative quenching following photoexcitation of a low-spin Fe­(II) polypyridyl complex gives rise to the situation depicted in Figure 5a (again, presented in terms of one-electron descriptions of the various electronic states involved for simplicity). Conversion from the initially formed ^1^MLCT excited state of the chromophore to the ^5^T_2_ ligand-field excited state—which represents the lowest-energy excited state in this class of compounds—occurs on a subpicosecond time scale.^32,87–89^ Accordingly, diffusion-based chemistry, such as dynamic quenching by a substrate, will involve this excited state of the chromophore which derives from a (t_2g_)^4^(e_g_*)^2^ configuration. Interaction between the excited Fe­(II) species and a suitable, diamagnetic acceptor (e.g., DDQ) will produce a transient donor–acceptor assembly characterized by *S*_T_ = 2. Transfer of a single electron from the e_g_* orbital set to the acceptor (Figure 5, path 1a) leaves Fe­(III) in an excited *S* = ^3^/_2_ state arising from (t_2g_)^4^(e_g_*)^1^ and a semiquinone; the presence of both *S*_T_ = 1 and *S*_T_ = 2 states for the donor–acceptor product assembly makes available a spin-allowed pathway, albeit with a reorganization energy associated with the one-electron change in σ-antibonding orbital population that will be roughly comparable to paths 1a and 2b of Co­(III) complexes with weak-to-moderate field ligands (Figure 4). However, whereas these steps for Co­(III) complexes account for essentially all of the reorganization energy required for product formation, there is a *two-electron* net change in σ-antibonding orbital population between the ^5^T_2_ excited state of Fe­(II) and the low-spin ground state of Fe­(III). One more step, namely conversion from the (t_2g_)^4^(e_g_*)^1^ *S* = ^3^/_2_ state (which corresponds to an excited electronic state) to the (t_2g_)^5^(e_g_*)^0^ low-spin Fe­(III) ground state, has to occur (Figure 5a, path 1b), so complete photoredox conversion from reactant to product for Fe­(II) polypyridyl-based chromophores is predicted to involve two barriers, each comparable in magnitude to the single barrier of the corresponding Co­(III) complex.

**Figure 5:**
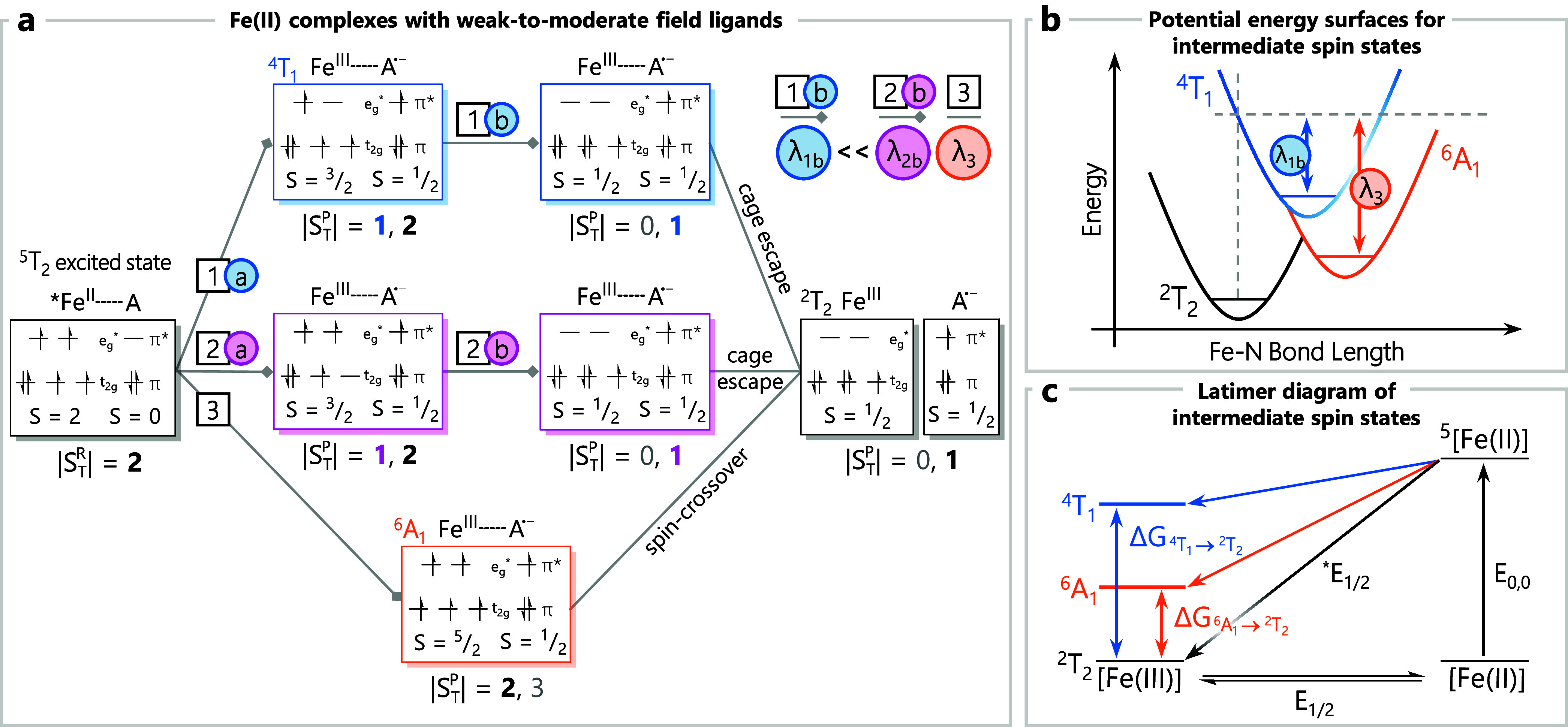
(a) Simplified consideration of spin selection rules for photoinduced electron transfer from the ^5^T_2_ excited state of Fe­(II) to an electron acceptor (A). Three spin allowed pathways are presented, forming Fe­(III), d^5^ intermediate spin states with ^4^T_1_ configuration (path 1, blue), (t_2g_)^3^(e_g_*)^2^ configuration with an electron pair in the t_2g_ set (path 2, pink), and ^6^A_1_ configuration (path 3, orange). Total spin of the reactant (| $S_{T}^{R}$ |) and the product (| $S_{T}^{P}$ |) was determined by considering the individual microspin states (S) for the cobalt species and donor as described in reference 80. (b) Potential energy surface diagram representing the energy difference between the ^6^A_1_ (orange) and ^4^T_1_ (blue) intermediate states and the ^2^T_2_ ground state (black), reflecting the large reorganization energy (λ) required to reach the ^2^T_2_ ground state. (c) Latimer diagram indicating free energy differences (Δ*G*) between the intermediate spin states and the final ^2^T_2_ state.

Two additional pathways can be envisioned stemming from the ^5^T_2_ excited state. Path 2 (pink boxes) in Figure 5a involves initial removal of an electron from the t_2g_ set, leaving a *S* = ^3^/_2_ state derived from the resulting (t_2g_)^3^(e_g_*)^2^ configuration. This is also an excited state of Fe­(III)—likely much higher in energy relative to the ground state than the *S* = ^3^/_2_ state sampled along path 1—that now must undergo conversion to the low-spin Fe­(III) configuration. This represents a two-electron transition but can be achieved along a spin-allowed pathway through the *S*_T_ = 1 states present in both the intermediate and ground states prior to cage escape (Figure 5a, path 2b). This stands in stark contrast to path 3, where initial removal of an electron from a doubly occupied t_2g_ orbital—yielding a *S* = ^5^/_2_ high-spin Fe­(III) species—cannot sample a spin-allowed pathway directly to the ground state of the system, but must instead couple to a *S* = ^3^/_2_ state (either via electronic mixing or in the form of an actual chemical intermediate) prior to ground-state formation. The second step of the conversion depicted in path 3 is equivalent to a spin-crossover event, which has been a known phenomenon for certain Fe­(III) complexes possessing ligand-field strengths comparable to the spin-pairing energy since the 1930s.^90^ Both of these pathways, i.e., paths 2 and 3 in Figure 5a, will necessarily involve reorganization energies twice that of the corresponding Co­(III) complexes, or roughly 10 000–12 000 cm^–1^.^31^

In addition to the significant barriers stemming from the large reorganization energies just described (Figure 5b), we hypothesize that spin-state limitations in forming the oxidized Fe­(III) product may also weaken the effective excited state oxidation potential accessible from the ^5^T_2_ state in Fe­(II), something that is not captured in the conventional Rehm–Weller analysis. While the zero-point energy (*E*_0,0_) remains unchanged regardless of the spin state of the photoproduct, the effective excited-state oxidation potential is governed by the initially formed Fe­(III) intermediate. From an electrochemical standpoint, oxidation of the Fe­(II) ^1^A_1_ ground state to form the ^4^T_1_ or ^6^A_1_ Fe­(III) states would require a significantly more positive potential than that needed to access the thermodynamically preferred ^2^T_2_ Fe­(III) state (Figure 5c). This redox potential shift effectively lowers the excited-state oxidation potential, thereby limiting the applicability of the Rehm–Weller equation, which assumes that the ^2^T_2_ Fe­(III) state is formed directly upon ET from the ^5^T_2_ ES.

Although the redox potentials associated with oxidation to the ^4^T_1_ or ^6^A_1_ Fe­(III) states cannot be measured experimentally, it is reasonable to assume that the higher-energy spin states are significantly less favorable than formation of the low-spin ^2^T_2_ state due to the population of one or two electrons in the antibonding e_g_* orbital. At minimum, the excited-state oxidation potential would be lowered by the Gibbs free energy difference (Δ*G*_0_) between the ^2^T_2_ and the intermediate ^4^T_1_ or ^6^A_1_ spin states (Figure 5c). We suggest that this overestimation of the effective ES oxidation potential ultimately limits the utility of the Rehm–Weller equation for accurately predicting the thermodynamic viability of Fe­(II) polypyridyl complexes as productive photoredox catalysts. This issue is particularly problematic given that the ^5^T_2_ excited state already stores relatively little energy for photochemical applications,^91^ which is further diminished by the formation of Fe­(III) spin-state intermediates.

### An Argument for Strong-Field First-Row-Based Chromophores for Photoredox

The analysis presented for the Co­(III) complexes with weak-to-moderate field ligands (Figure 4)—in particular, the mechanistic aspects of path 2—highlights an interesting scenario for the case of Co­(III) complexes composed of ligands capable of stabilizing the low-spin form of Co­(II) as its ground state. The situation is illustrated in Figure 6a. Starting from the same point as in Figure 4, electron transfer from the substrate to the ^3^T_1_ excited state of Co­(III) can go via the same two pathways as described before. Path 1, as in Figure 4, involves the formation of high-spin Co­(II) which now must undergo a spin-flip to the (t_2g_)^6^(e_g_*)^1^ configuration that characterizes the ground state of low-spin Co­(II). This represents the microscopic reverse of the corresponding spin-flip of path 2 in Figure 4 and should therefore be defined by comparable values for its inner-sphere reorganization energy. In contrast, electron transfer along path 2 in the strong-field case directly leads to the product (Figure 6a). The key difference here is that the energetic cost of this process—which is essentially what the reorganization energy reflects—is now only associated with the addition of the electron to the t_2g_ orbital set. The bulk of the reorganization energy, which in this case is defined by the single occupancy of the e_g_* orbitals, has already been absorbed by photoexcitation and thermalization of the ^3^T_1_ excited state of Co­(III). This leads to the conclusion that Co­(III) complexes comprised of ligands such as carbenes, whose ligand-field strengths can stabilize low-spin Co­(II), should give rise to significantly different kinetic profiles for photoredox catalysis than what has been observed for Co­(III) polypyridyls due to a substantial reduction in λ_i_.

**Figure 6:**
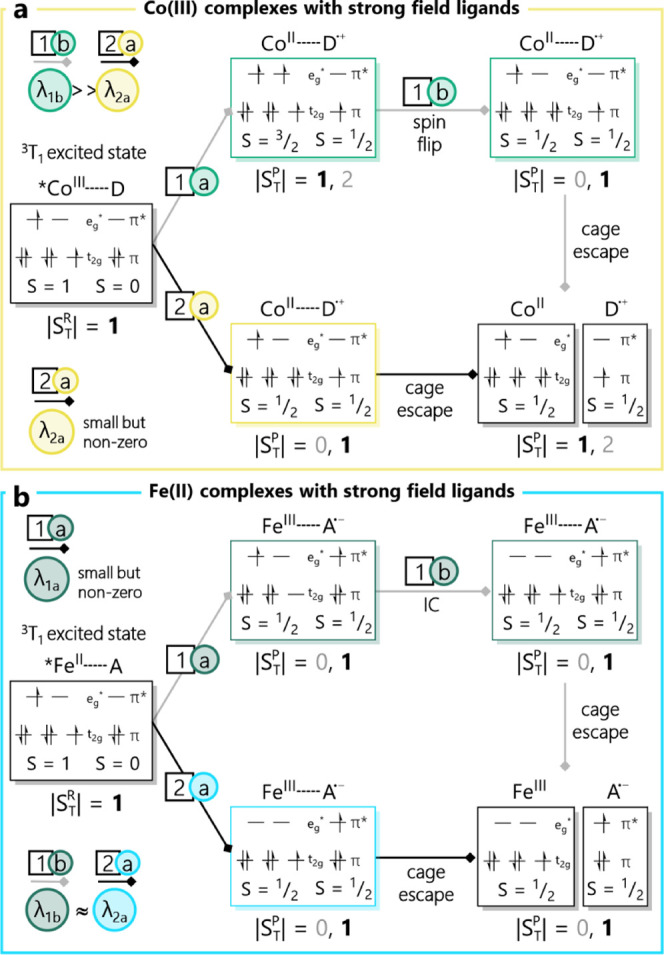
(a) Simplified consideration of spin selection rules for photoinduced electron transfer from a donor (D) to the ^3^T_1_ excited state of Co­(III) complexes containing strong field ligands. (b) Simplified consideration of spin selection rules for photoinduced electron transfer to an electron acceptor (A) from the ^3^T_1_ excited state of Fe­(II) complexes containing strong field ligands.

Turning back to the case of Fe­(II), the situation depicted in Figure 5 is endemic for oxidative quenching of the ^5^T_2_ excited state of a d^6^ configuration metal complex characterized by a ^1^A_1_ ground state (e.g., Fe­(II) polypyridyls). Recently, researchers have started to develop strong-field ligands via the incorporation of carbene functionalities into the primary coordination sphere of the metal center.^55,72,73,92–101^ These have resulted in several examples for which the lowest energy excited state of the Fe­(II) complex appears to be the ^3^T_1_ state instead of the ^5^T_2_ characteristic of polypyridyl complexes. For this emerging class of chromophores, oxidative quenching yields the two possible reaction trajectories shown in Figure 6b. Removal of an electron from the lower energy t_2g_ set (path 1) yields a *S* = ^1^/_2_ state that represents an excited electronic state of low-spin Fe­(III) (i.e., a (t_2g_)^4^(e_g_*)^1^ configuration). The association of this species with the substrate radical formed via electron transfer allows for a spin-allowed pathway by virtue of the *S*_T_ = 0 and *S*_T_ = 1 states that will be present. Product formation involves conversion of the Fe­(III)-based excited state to its (t_2g_)^5^(e_g_*)^0^ ground state and will be present with a reorganization energy comparable to that expected for the ground-state recovery step of path 1 in Figure 5a. The real advantage of leveraging the ^3^T_1_ excited state of an Fe­(II) complex is made apparent by examining path 2 (Figure 6b). Oxidative quenching of the ^3^T_1_ excited state via removal of the e_g_* orbital results in the immediate formation of the ground-state configuration of the low-spin Fe­(III) photoproduct. The situation is directly analogous to that depicted in path 1 of Figure 5a, where the structural reorganization of the entire pathway is completely comprised of formation and thermalization of the ^3^T_1_ excited state of the chromophore before the electron transfer event occurs. As with Co­(III) complexes whose ligand environment can stabilize low-spin Co­(II), Fe­(II) chromophores for which the ^3^T_1_ state is the lowest energy excited state should be expected to be particularly versatile for applications in photoredox catalysis.

## Concluding Comments

The initial goal of this effort was to expand on previously published results suggesting that the ^5^T_2_ excited state of Fe­(II) polypyridyl complexes can be leveraged for photoinduced electron transfer. Specifically, we sought to employ a range of Fe­(II) polypyridyl complexes possessing stronger ligand fields that would store more energy in the excited state and/or increase the driving force for electron transfer. A lack of reactivity from these compounds despite more favorable conditions for electron transfer prompted us to reexamine our prior work. In so doing, we were able to reproduce the findings from that initial communication, i.e., the *appearance* of dynamic quenching of the Fe­(II)-based excited state, but were able to trace its origin to a previously unidentified reaction between the ground states of [Fe­(tren­(py)_3_)]^2+^ and the benzoquinone-based acceptors that led to a reduction in the lifetime of the ^5^T_2_ state of [Fe­(tren­(py)_3_)]^2+^. While this result made the lack of reactivity for other Fe­(II) polypyridyls less surprising, it raised an entirely new question: why, despite what appeared to be favorable conditions for photoinduced oxidative quenching of the ^5^T_2_ excited state of the Fe­(II)-based chromophores, was there no reaction occurring? This result stands in stark contrast to behavior observed for Co­(III) polypyridyl complexes, where reductive quenching of its ligand-field excited state(s) is well established.

A detailed analysis of the reaction pathways available for electron transfer involving metal-centered, ligand-field excited states revealed that the likely origin of these differences in reactivity stem from spin-state and reorganization energy parameters that uniquely arise when dealing with electron transfer involving ligand-field excited states. Our analysis revealed what we believe is a fundamental limitation of ^5^MC states, where ET from the Fe­(II) ^5^T_2_ excited state requires population of intermediate spin states and large structural reorganizations that impose significant energetic and spin-related barriers to productive photoredox catalysis. In contrast, the limitations inherent to ^5^T_2_ states are lifted in ^3^T_1_ MC excited states. For example, Co­(III) complexes with weak-to-moderate ligand fields can undergo photoinduced ET through spin-allowed pathways, directly yielding high-spin Co­(II) photoproducts with substantially reduced reorganization costs, enabling broader photoredox applicability.^16,33,102^ This analysis also revealed that reactivity can be recovered for Fe­(II) complexes that possess sufficiently strong ligand fields (e.g., carbenes) that can stabilize its ^3^T_1_ state as its lowest-energy excited state.^93,94,96,98,72,73,103,104^ In these systems, photoinduced ET can directly yield low-spin Fe­(III) photoproducts due to reduced structural reorganization as compared to what is required for reactions involving the ^5^T_2_ excited state. Although short picosecond lifetimes often limit the applicability of ^3^T_1_ excited states in strong-field Fe­(II) systems, strategies including ligand design^71,105^ and preassociation^106–111^ offer promising avenues to overcome these challenges. Moreover, these strong-field ligand environments also benefit Co­(III) photocatalysts by increasing the ligand field splitting in the Co­(II) product state, which favors the low-spin photoproduct upon ET from a donor. Compared to the high-spin Co­(II) species produced with weak-to-moderate ligand field strength, the low-spin pathway requires significantly smaller structural changes upon electron transfer. Consequently, such systems combine several advantages including decay pathways that operate in the Marcus inverted region, allowing for higher excited-state energies,^71^ longer excited-state lifetimes,^33,61,112^ and reduced reorganization barriers required for photoredox transformations.

Overall, these findings highlight the influence of excited-state spin multiplicity and reorganization energy as key factors that are endemic to metal-center-based photoredox reactivity, particularly in complexes of the first transition series where ligand-field excited states play a dominant role in their photophysics. With continued advances in ligand design and excited-state control, we would argue that, with proper consideration of factors that are largely overlooked, first-row transition metal complexes hold significant promise as efficient, selective, and sustainable chromophores for photoredox catalysis.

## Supplementary Material



## Notes

Experimental details, spectroscopic characterization, electrochemistry, and optical measurements, including UV–vis and TA spectroscopy (PDF)
