# Supplementary material for: Spin-State and Reorganization Energy Considerations for Metal-Centered Photoredox Catalysis
Source: J Am Chem Soc. 2025 Oct 16;147(43):39898–911. doi: 10.1021/jacs.5c14935 (PMC12576781; doi:10.1021/jacs.5c14935)
Supplement: Supplementary file 1 [file ja5c14935_si_001.pdf]

Supporting Information

Spin-State and Reorganization Energy Considerations for Metal-Centered Photoredox Catalysis

Bekah E. Bowers,[a] Björn Pfund,[a] Hayden F. Beissel, Atanu Ghosh, and James K. McCusker*

Department of Chemistry, Michigan State University, East Lansing, Michigan, 48824, USA

Correspondence to: jkm@chemistry.msu.edu

[a] B.E.B and B.P contributed equally to this work.

Table of Contents

1. **Experimental Details** S3
   - *i. General* S3
   - *ii. UV-Vis Spectroscopy* S3
   - *iii. Electrochemistry* S3
   - *iv. Transient Absorption Spectroscopy* S4
2. **Photochemical Properties of Fe(II) Polypyridyl Complexes** S5
   - *i. Photochemical Properties of* $[Fe(bpy)_3]^{2+}$ S5
   - *ii. Photochemical Properties of* $[Fe(terpy)_2]^{2+}$ S6
   - *iii. Photochemical Properties of* $[Fe(4'\text{-}OMe\text{-}terpy)_2]^{2+}$ S7
3. **Ground State Reactivity of** $[Fe(tren(py)_3)]^{2+}$ S8
   - *i.* ^{1}H *NMR Spectra of Decomposition* S8
   - *ii. UV-Vis Transient Absorption Spectroscopy of Decomposition* S14
4. **Ligand Field Energetics of Co(III) Complexes** S15
5. **Expanded Spin-State Analysis** S16
6. **References** S17

1. Experimental Details

i. General

The following starting materials were purchased from Ambeed and used without further purification: 2,2'-bipyidine (bpy), 2,2':6'2''-terpyridine (terpy), and 4'-chloro-2,2':6'2''-terpyridine. 4'-Methoxy-2,2':6',2''-terpyridine (4'-OMe-terpy) was synthesized according to literature procedure[1] by reaction of sodium methoxide in methanol with 4'-chloro-2,2':6'2''-terpyridine. Anhydrous $FeCl_2$ was purchased from Strem and used as received. The following complexes were synthesized using literature procedures: $[Fe(tren(py)_3)](PF_6)_2$,[2] $[Fe(bpy)_3](PF_6)_2$,[3] $[Fe(terpy)_2](PF_6)_2$,[4] and $[Fe(4'\text{-OMe-terpy})_2](PF_6)_2$.[1] 2,3-dichloro-5,6-dicyano-1,4-benzoquinone (DDQ) was obtained from Oakwood Chemicals, respectively, and recrystallized twice from dichloromethane. 3,4,5,6-Tetrachloro-*ortho*-benzoquinone (*o*-TCBQ) and 2,3,5,6-tetrachloro-*para*-benzoquinoe (*p*-TCBQ) was obtained from Sigma Aldrich and recrystallized from dichloromethane. 3,4,5,6-Tetrabromo-*ortho*-benzoquinone (*o*-TBBQ) was synthesized according to literature procedure.[5] Reagent grade solvents were obtained from Sigma-Aldrich and used as received. Spectrophotometric-grade acetonitrile (Fisher Chemical) was used for all steady-state and time-resolved measurements and used as received. All solvents from the Argon filled glovebox were freeze-pump-thawed before use in spectroscopic measurements. ^1H NMR spectra were recorded in d_3-MeCN on a 500 MHz Agilent DDR2 spectrometer at Max T. Roger NMR facility, Michigan State University. All NMR spectra were processed using Mestrenova, and chemical shifts were referenced to the residual solvent signal (^1H NMR: δ = 1.96 ppm and ^{13}C NMR: δ = 118.26 ppm for CD_3CN).[6] Electrospray ionization mass spectrometry (ESI-MS) spectra acquired in HPLC-grade acetonitrile on a Waters G2-X2 QToF mass analyzer at the Mass Spectrometry and Metabolomics Core Facility, Michigan State University.

ii. UV-Vis Spectroscopy

UV-Vis absorption spectra were recorded at room temperature on a Varian Cary 50 or PerkinElmer Lambda 1050 spectrophotometer in 1 cm path length quartz cuvette and spectrophotometric-grade acetonitrile.

iii. Electrochemistry

Electrochemical measurements were collected using a CH Instrument model CH1620D electrochemical workstation on nitrogen purged solutions. A standard three-electrode setup consisting of a Pt-disc working electrode, a silver wire pseudo-reference electrode, and a platinum wire counter electrode in a MeCN solution containing 0.1 M tetra-*n*-butylammonium hexafluorophosphate ($TBAPF_6$) as a supporting electrolyte. $TBAPF_6$ was purchased from Oakwood Chemicals and recrystallized twice from ethanol before use. All measurements were internally referenced to the Fc/Fc^+ redox couple.

Cyclic voltammetry experiments were conducted at a scan rate of 100 mV/s and differential pulse voltammetry was conducted at a scan rate of 4 mV/s.

iv. Transient Absorption Spectroscopy

Ultrafast experiments (Fe(II)-complex lifetimes less than 10 ns) were performed on a home-built laser system operating at 5.0 W and providing 400 mW (76 MHz) mode-locked beam as the seed for Ti:sapphire regenerative amplifier. The output (800 nm, 1 KHz) from the amplifier is split into the pump and probe lines at 70:30 ratio, respectively. The pump wavelength was generated from an optical parametric amplifier (OPA) and the probe beam was generated from a white light continuum emerging from a sapphire crystal. Data were collected at room temperature (20°C) in either 1 mm or 1 cm quartz cuvettes.

Nanosecond transient absorption data (ns-TA) were acquired using an Edinburgh Instruments LP980 spectrometer fitted with a Hamamatsu R928 photomultiplier tube and interfaced to a Tektronix TDS 3032C oscilloscope. An Opotek Vibrant 355 LB Q-switched Nd:YAG laser was used as an excitation source. All data was collected at 20°C using a Quantum Northwest Brr 30 peltier temperature-controlled cuvette holder in a 1 cm quartz cuvette.

All samples for transient absorption were prepared in an Ar-filled dry box with freeze-pump-thawed spectroscopic grade MeCN. Stock solutions of Fe(II)-complexes were prepared (0.05 mM – 0.1 mM concentrations) and added to volumetric flasks containing different amounts of quencher to produce solutions of known concentration. UV-Vis spectra were collected before and after TA measurements to ensure no sample decomposition had occurred. All data were processed using IgorPro software by Wavemetrics.

2. Photochemical Properties of Fe(II) Polypyridyl Complexes

i. Photochemical Properties of [Fe(bpy)3]2+

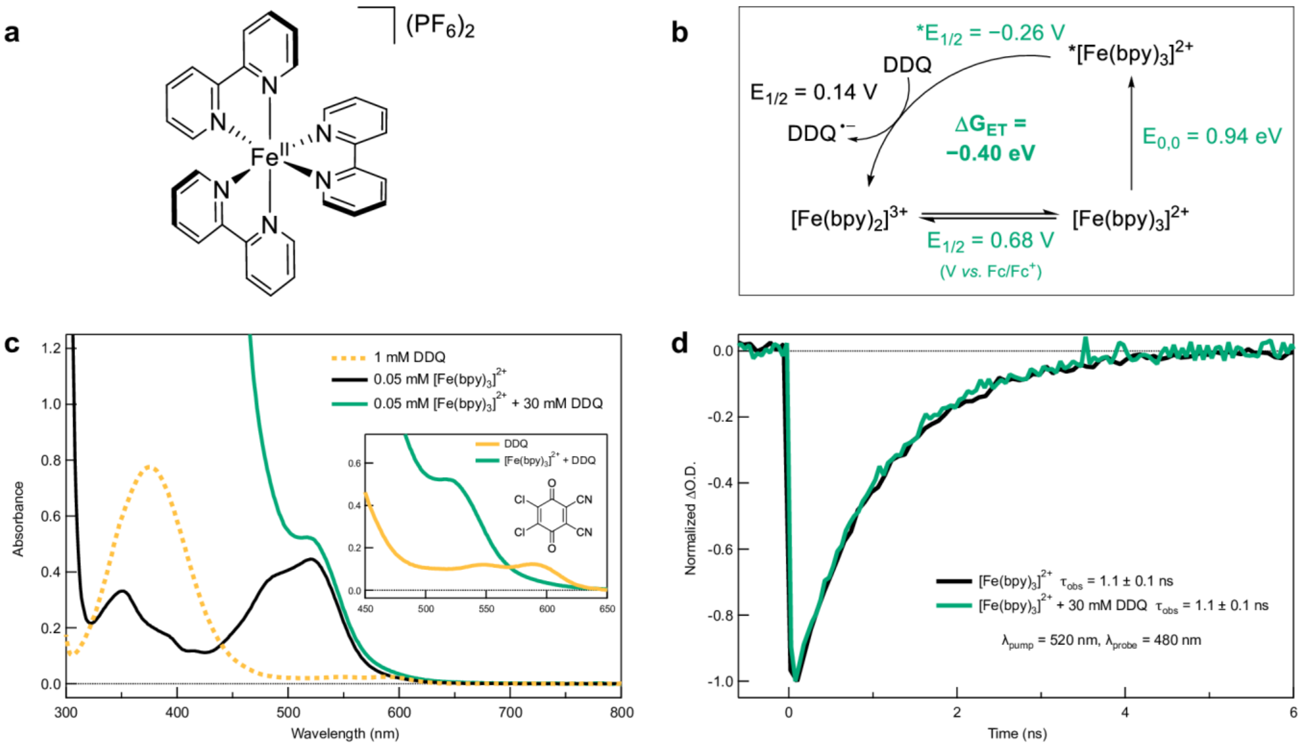

Figure S1. (a) Chemical structure of $[Fe(bpy)_3](PF_6)_2$. (b) Latimer diagram for the oxidative quenching cycle of $[Fe(bpy)_3]^{2+}$ based on previously reported ground state redox potentials ($E_{1/2}$) and zero-point energy ($E_{0,0}$) of the $^5T_2$ excited state.[7] This yields an excited stated redox potential ($*E_{1/2}$) sufficient to reduce DDQ with a driving force (ΔG_{ET}) of −0.4 eV. (c) UV-Vis absorption spectra of $[Fe(bpy)_3]^{2+}$, DDQ, and Fe-complex with 30 mM DDQ mixture in MeCN. Inset: UV-Vis absorption spectra of DDQ band from 450 to 650 nm compared to $[Fe(bpy)_3]^{2+}$ MLCT band. (d) Normalized single wavelength transient absorption kinetics of $[Fe(bpy)_3]^{2+}$ and $[Fe(bpy)_3]^{2+}$ with 30 mM DDQ in MeCN at 20 °C probed at 480 nm following excitation at 520 nm.

ii. Photochemical Properties of [Fe(terpy)$_2$]$^{2+}$

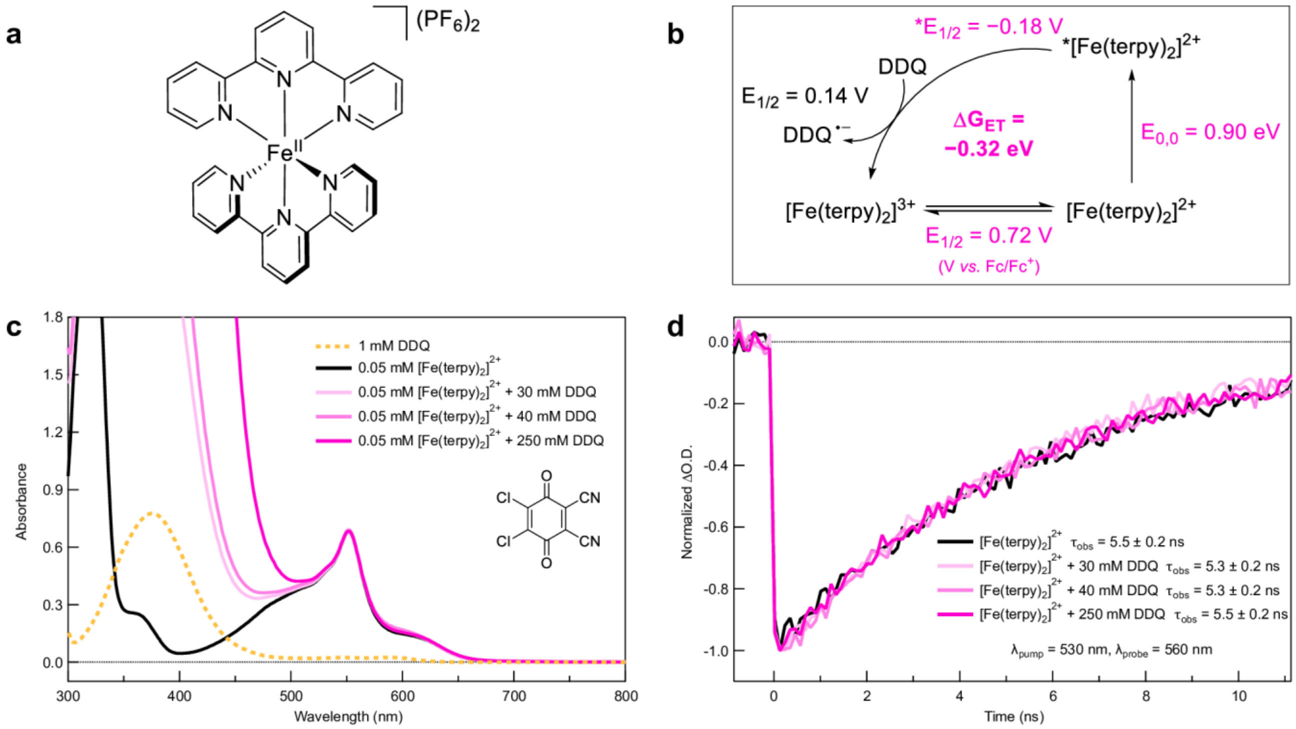

Figure S2. (a) Chemical structure of [Fe(terpy)$_2$](PF$_6$)$_2$. (b) Latimer diagram for the oxidative quenching cycle of [Fe(terpy)$_2$]$^{2+}$ based on previously reported ground state redox potentials ($E_{1/2}$) and zero-point energy ($E_{0,0}$) of the $^5T_2$ excited state.[7] This yields an excited stated redox potential (*$E_{1/2}$) sufficient to reduce DDQ with a driving force (ΔG_{ET}) of −0.32 eV. (c) UV-Vis absorption spectra in MeCN of [Fe(terpy)$_2$]$^{2+}$, DDQ, and Fe-complex with 30 mM, 40 mM, and 250 mM DDQ mixture. (d) Normalized single wavelength transient absorption kinetics of [Fe(terpy)$_2$]$^{2+}$ and Fe-complex with 30 mM, 40 mM, and 250 mM DDQ in MeCN at 20 °C, probed at 560 nm following excitation at 530 nm.

iii. Photochemical Properties of [Fe(4’-OMe-terpy)$_2$]$^{2+}$

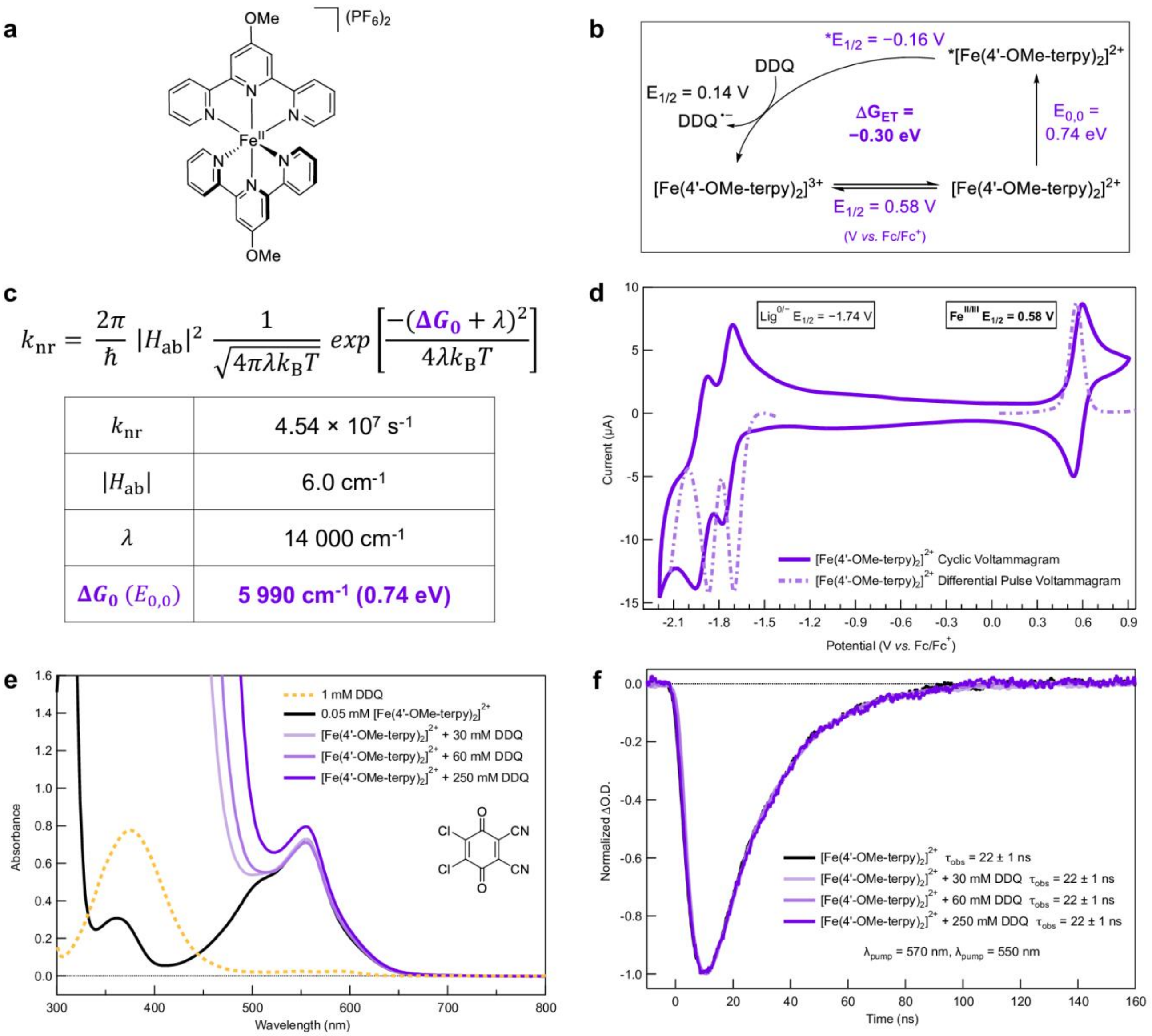

Figure S3. (a) Chemical structure of [Fe(4’-OMe-terpy)$_2$](PF$_6$)$_2$. (b) Latimer diagram for the oxidative quenching cycle of [Fe(4’-OMe-terpy)$_2$]$^{2+}$ based on zero-point energy ($E_{0,0}$, estimated in Figure SXXXc) and ground state redox potential ($E_{1/2,}$ Figure SXXXd) of the $^5T_2$ excited state.[7] This yields an excited stated potential (*$E_{1/2}$) sufficient to reduce DDQ with a driving force (ΔG_{ET}) of −0.30 eV. (c) Semi-classical Marcus theory equation and its parameters for determining the zero-point energy ($E_{0,0}$) of the $^5T_2$ excited state. The nonradiative rate (k_{nr}) was determined via transient absorption kinetics, and the electronic coupling (H_{ab}) and reorganization energy (λ) values are based on reported values for [Fe(terpy)$_2$]$^{2+}$.[7] (d) Cyclic voltammogram and differential pulse voltammogram for [Fe(4’-OMe-terpy)$_2$]$^{2+}$ in MeCN using 0.1 M TBAPF$_6$ as a supporting electrolyte. (e) UV-Vis absorption spectra in MeCN of [Fe(4’-OMe-terpy)$_2$]$^{2+}$, DDQ, and Fe-complex with 30 mM, 60 mM, and 250 mM DDQ mixture. (f) Normalized single wavelength transient absorption kinetics of [Fe(4’-OMe-terpy)$_2$]$^{2+}$ and Fe-complex with 30 mM, 60 mM, and 250 mM DDQ in MeCN at 20 °C, probed at 550 nm following excitation at 570 nm.

3. Ground State Reactivity of $[Fe(tren(py)_3)]^{2+}$

i. ^{1}H NMR Spectra of Decomposition

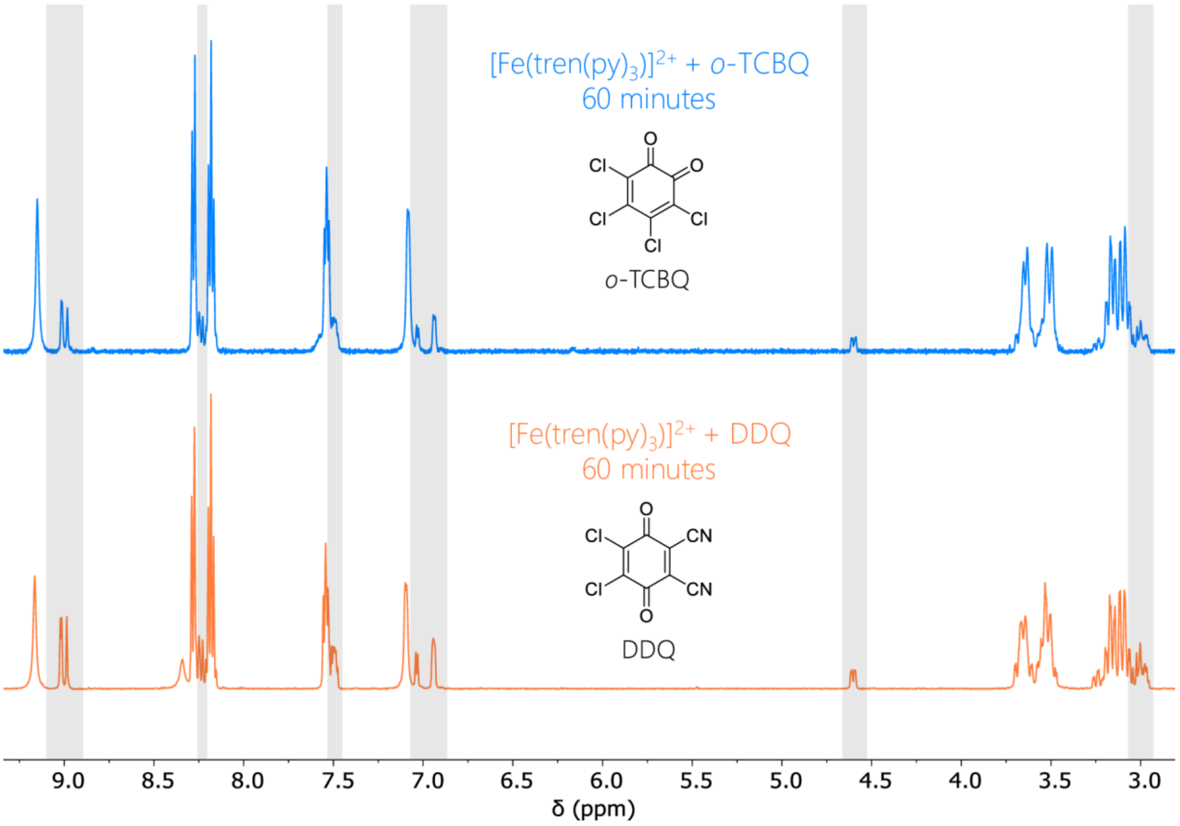

Figure S4. Stacked ^{1}H NMR for solutions in d_3-MeCN of $[Fe(tren(py)_3)]^{2+}$ with *o*-TCBQ (top, blue) and $[Fe(tren(py)_3)]^{2+}$ with DDQ (bottom, orange) after 60 minutes of mixing showing the growth of the same Fe(II) decomposition product highlighted in grey.

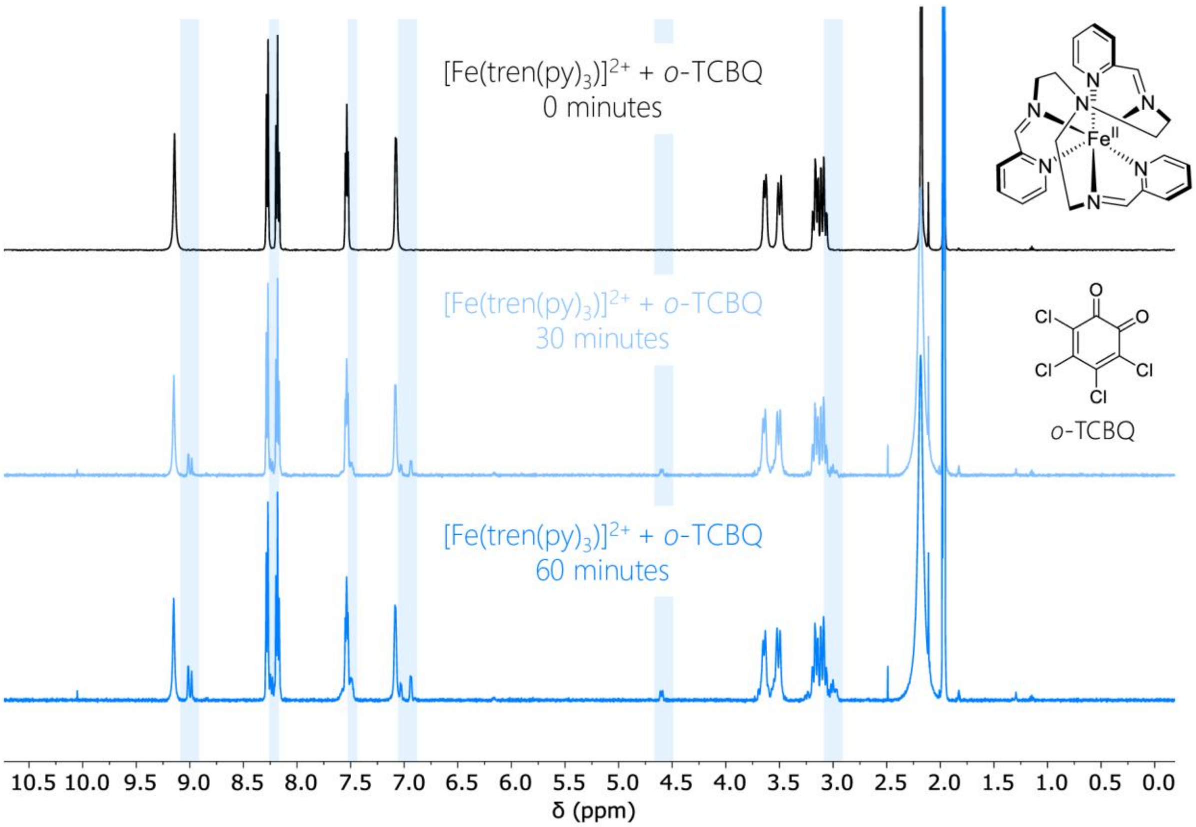

Figure S5. ^{1}H NMR in d_3-MeCN for $[Fe(tren(py)_3)]^{2+}$ with *o*-TCBQ over time with initial mixing (top, black), 30 minutes after mixing (middle, light blue), and 60 minutes after mixing (bottom, dark blue) showing the increasing concentration of decomposition product over time (highlighted in blue).

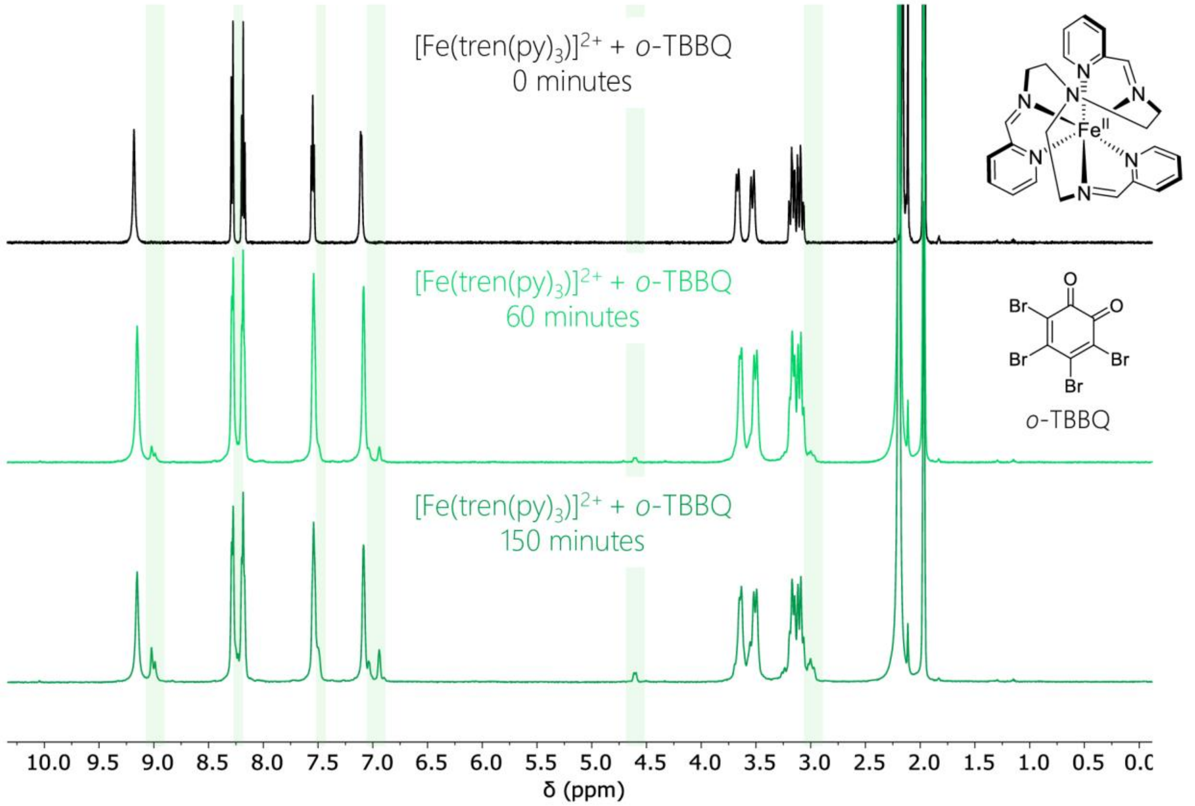

Figure S6. ^{1}H NMR in d_3-MeCN for $[Fe(tren(py)_3)]^{2+}$ with *o*-TBBQ over time with initial mixing (top, black), 60 minutes after mixing (middle, light green), and 150 minutes after mixing (bottom, dark green) showing the increasing concentration of decomposition product over time (highlighted in green). The rate of decomposition is much slower with *o*-TBBQ compared to DDQ and *o*-TCBQ.

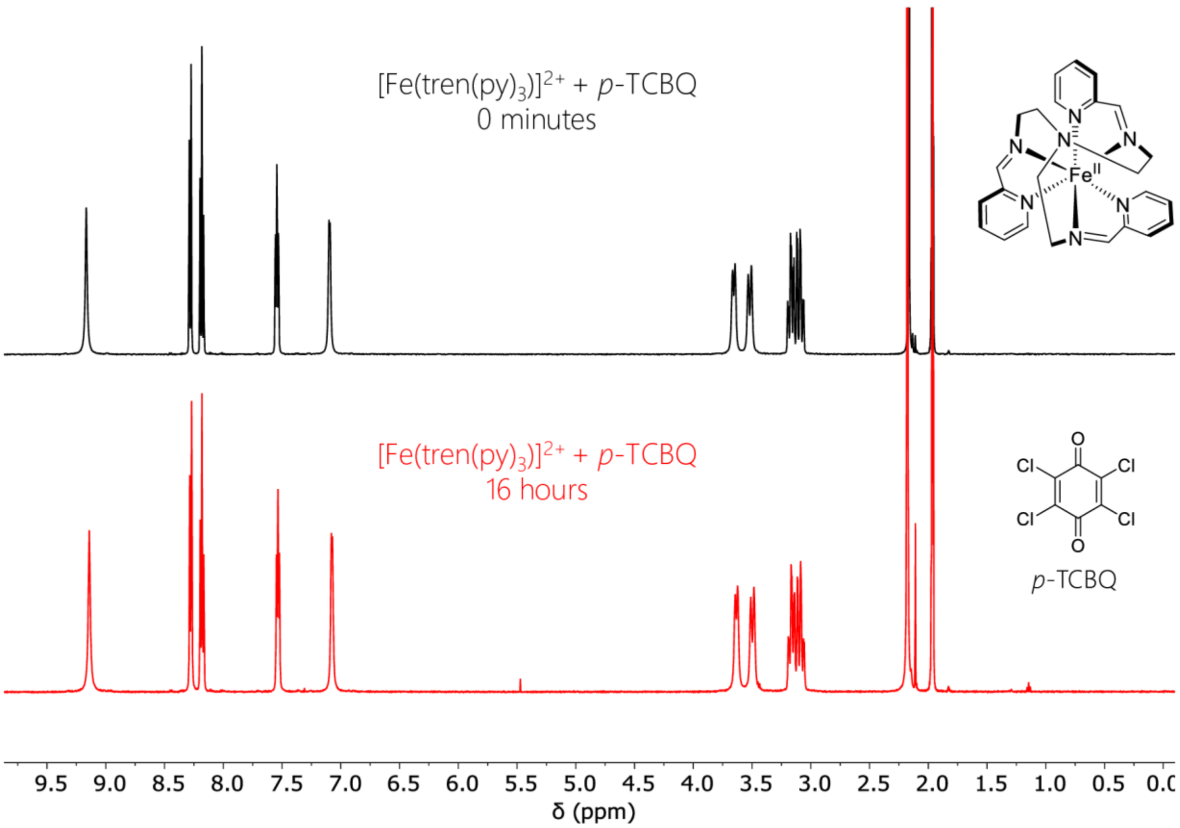

Figure S7. ^{1}H NMR in d3-MeCN for $[Fe(tren(py)_3)]^{2+}$ with *p*-TCBQ over time with initial mixing (top, black) and overnight mixing (bottom, red) showing no decomposition of the Fe-complex over 16 hours.

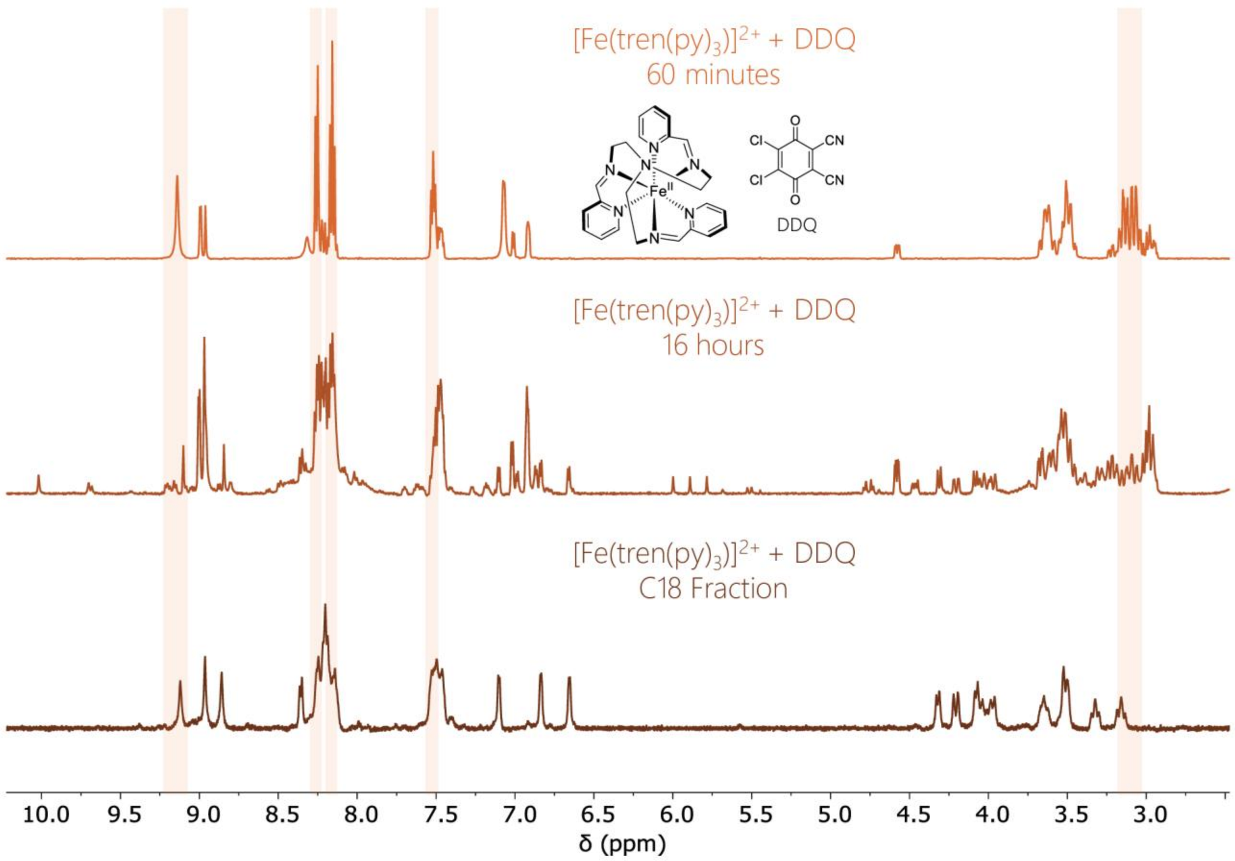

Figure S8. ^{1}H NMR in d_3-MeCN for $[Fe(tren(py)_3)]^{2+}$ with DDQ over time with 60 minutes after mixing (top, light orange), 16 hours after mixing (middle, dark orange), and after separation by reversed-phase column chromatography (bottom, brown). The signals highlighted in orange show the disappearance of the parent $[Fe(tren(py)_3)]^{2+}$ complex after reaction overnight. The separated decomposition product could not be identified by NMR or mass spectroscopy.

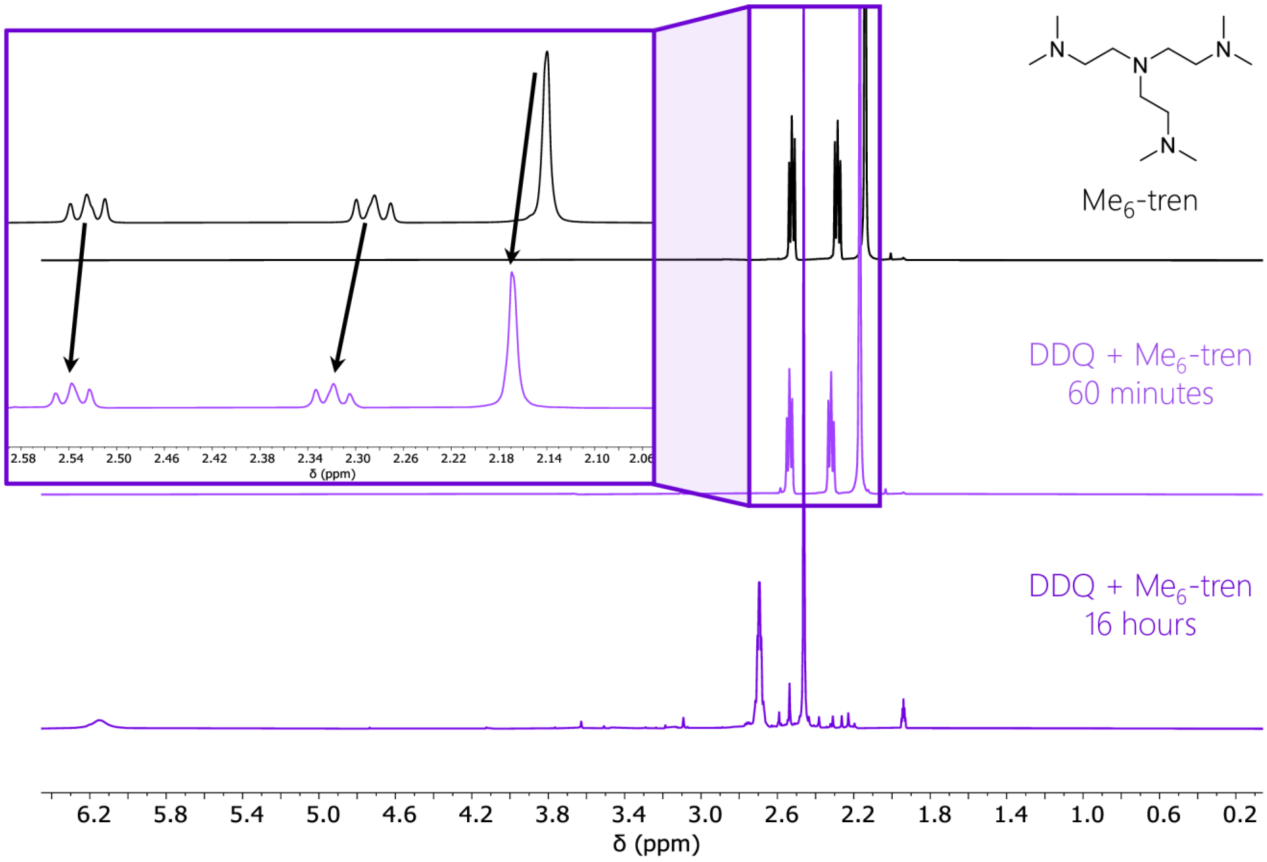

Figure S9. ^{1}H NMR in d_3-MeCN of Me_6-tren (top, black), DDQ and Me_6-tren mixture after 60 minutes (middle, light purple), and DDQ and Me_6-tren mixture after 16 hours (bottom, dark purple). Inset: Aliphatic region from 2.0 to 2.6 ppm showing the 0.03 ppm shift in the methyl and ethylene bridge resonances upon addition of DDQ.

ii. UV-Vis Transient Absorption Spectroscopy of Decomposition

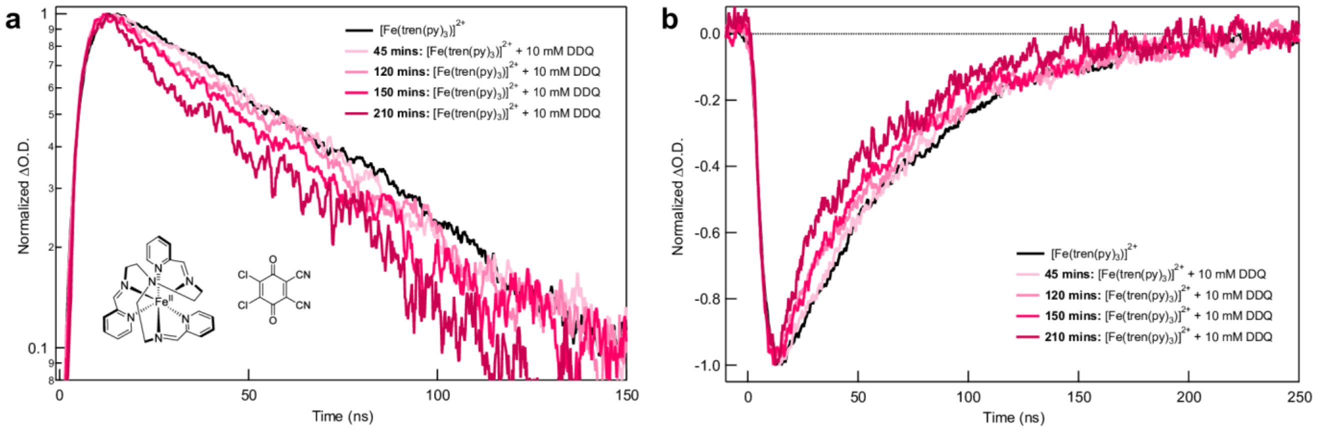

Figure S10. (a) Log plot of transient absorption kinetics at 560 nm following 580 nm excitation, at different time points after mixing (0, 30, 60, 120, 180 minutes) a sample containing 0.05 mM $[Fe(tren(py)_3)]^{2+}$, 10 mM DDQ, and 0.1 M $TBAPF_6$ in deaerated MeCN. (b) Transient absorption kinetics at 560 nm following 580 nm excitation plotted linearly, at different time points after mixing (0, 30, 60, 120, 180 minutes) a sample containing 0.05 mM $[Fe(tren(py)_3)]^{2+}$, 10 mM DDQ, and 0.1 M $TBAPF_6$ in deaerated MeCN.

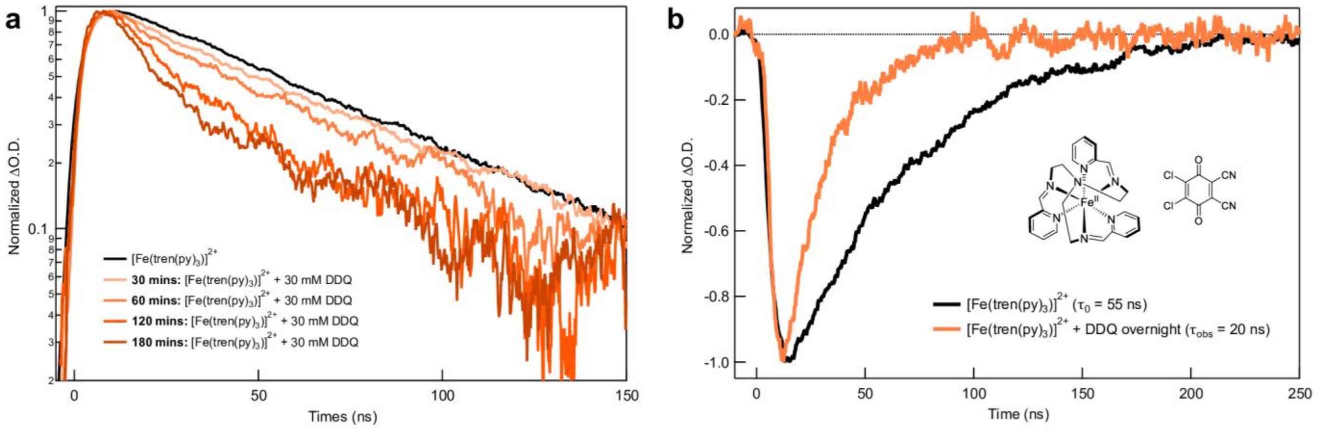

Figure S11. (a) Log plot of transient absorption kinetics at 560 nm following 580 nm excitation, at different time points after mixing (0, 30, 60, 120, 180 minutes) a sample containing 0.05 mM $[Fe(tren(py)_3)]^{2+}$, 30 mM DDQ, and 0.1 M $TBAPF_6$ in deaerated MeCN. A deviation from linearity is observed at longer mixing time points (120 and 180 minutes), indicating the emergence of two components. (b) Transient absorption kinetics in MeCN of fully decomposed product at 560 nm following 580 nm excitation on sample allowed to react overnight. A decrease in lifetime from 55 ns (parent $[Fe(tren(py)_3)]^{2+}$ complex) to 20 ns is observed.

4. Ligand Field Energetics of Co(III) Complexes

UV-Vis absorption data for $[Co(MeImP)_2]^+$ (MeImP = 1,1′-(1,3-phenylene)bis(3-methyl-1-imidazole-2-ylidene)) was extracted from Krishna *et. al.*[8] where the spin allowed $^1A_1 \rightarrow {}^1T_1$ absorption band centered around 27 700 cm^{-1} was fit using Gaussian deconvolution in IgorPro to determine the transition energy of the ligand field band.[9] The higher energy band beyond 29 000 cm^{-1} is associated with the $^1A_1 \rightarrow {}^1LMCT$ absorption band, the band centered around 28 700 cm^{-1} is associated with the second spin allowed $^1A_1 \rightarrow {}^1T_2$ ligand field band, and the band at 27 700 cm^{-1} is associated with the lowest spin allowed $^1A_1 \rightarrow {}^1T_1$ ligand field band.

a

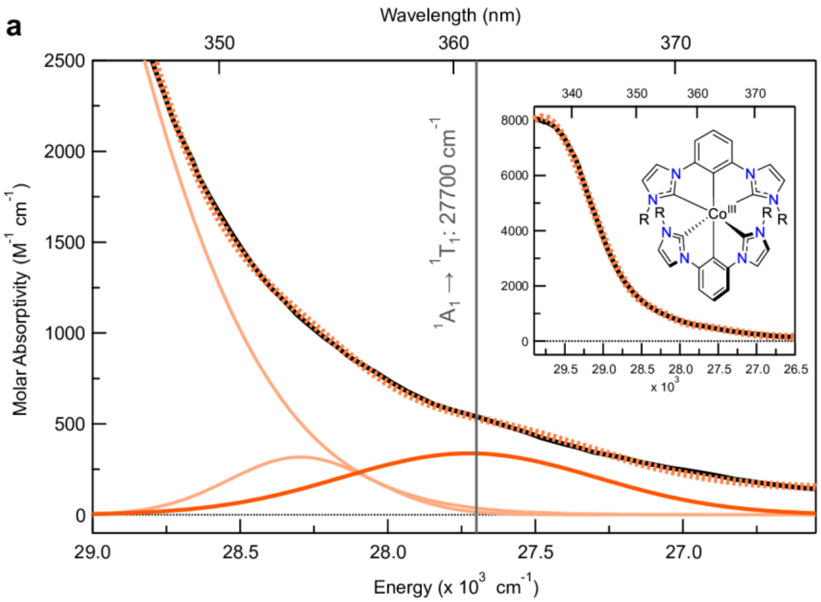

b

Complex	$^1A_1 \rightarrow {}^1T_1$ Energy (cm^{-1})
$[Co(4,4'\text{-}Br_2\text{-}bpy)_3]^{3+}$	21670
$[Co(MeImP)_2]^+$	27700

Figure S12. (a) UV-Vis absorption spectrum of $[Co(MeImP)_2]^+$ in MeCN extracted from Krishna *et. al.*[8] with ligand field bands (solid orange lines) fitted using Gaussian deconvolution, showing the lowest spin allowed $^1A_1 \rightarrow {}^1T_1$ ligand field band at 27 700 cm^{-1}. The orange dashed line is the fit function for all Gaussian peaks. The inset is the reported spectrum between 325 to 380 nm, showing the $^1A_1 \rightarrow {}^1LMCT$ absorption band. (b) $^1A_1 \rightarrow {}^1T_1$ transition energies for $[Co(4,4'\text{-}Br_2\text{-}bpy)_3]^{3+}$ [10] and $[Co(MeImP)_2]^+$.

5. Expanded Spin-State Analysis

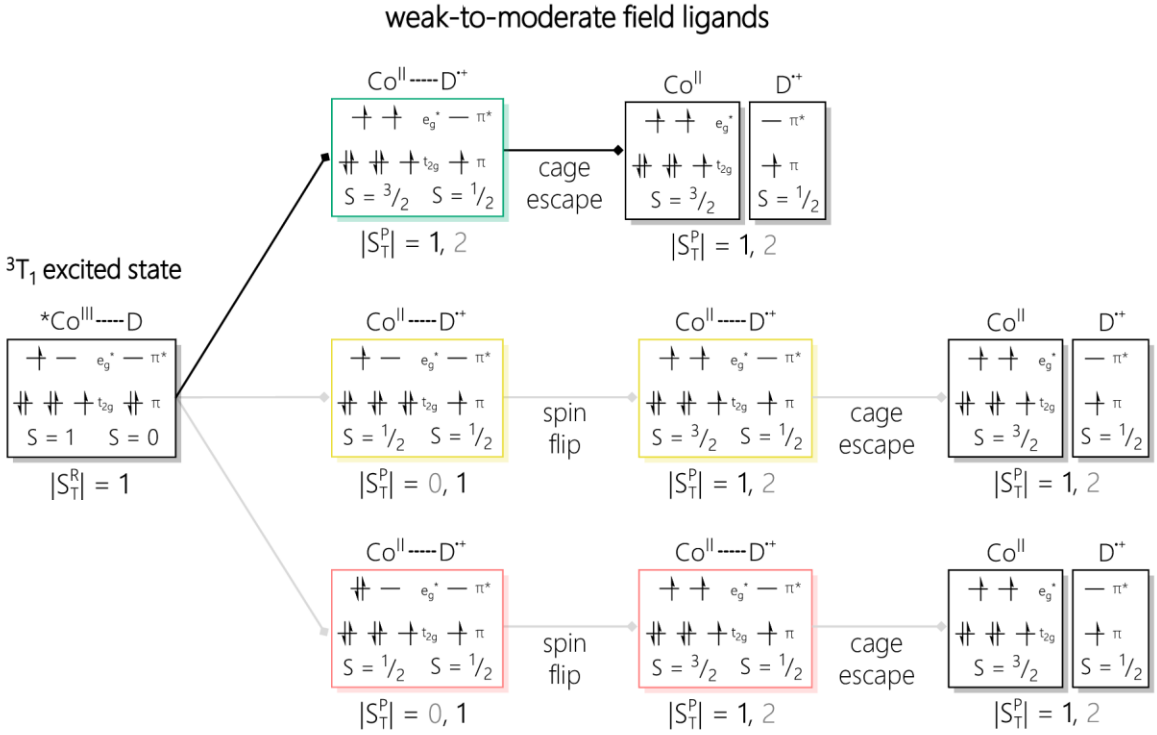

Figure S13. Simplified consideration of spin selection rules for photoinduced electron transfer from a donor (D) to the $^3T_1$ excited state of Co(III) complexes containing weak-to-moderate field ligands. Total spin of the reactant ($|S_T^R|$) and the product ($|S_T^P|$) was determined by considering the individual micro-spin states (S) for the cobalt species and donor. The green and yellow pathways were presented in the main text. The red pathway represents the third spin-allowed possibility where an electron is added to the partially filled e_g^* orbital and is less likely to be the dominant pathway compared to the above pathways.

6. References

1. Chambers, J.; Eaves, B.; Parker, D.; Claxton, R.; Ray, P. S.; Slattery, S. J. Inductive Influence of 4′-Terpyridyl Substituents on Redox and Spin State Properties of Iron(II) and Cobalt(II) Bis-Terpyridyl Complexes. *Inorg. Chim. Acta.* **2006**, *359*, 2400–2406.

2. Conti, A. J.; Xie, C. L.; Hendrickson, D. N. Tunneling in Spin-State Interconversion of Ferrous Spin-Crossover Complexes. Concentration Dependence of Apparent Activation Energy Determined in Solution by Laser-Flash Photolysis. *J. Am. Chem. Soc.* **1989**, *111*, 1171–1180.

3. Benazzi, E.; Cristino, V.; Caramori, S.; Meda, L.; Boaretto, R.; Bignozzi, C. A. Electrochemical Characterization of Polypyridine Iron(II) and Cobalt(II) Complexes for Organic Redox Flow Batteries. *Polyhedron* **2018**, *140*, 99–108.

4. Machan, C. W.; Adelhardt, M.; Sarjeant, A. A.; Stern, C. L.; Sutter, J.; Meyer, K.; Mirkin, C. A. One-Pot Synthesis of an Fe(II) Bis-Terpyridine Complex with Allosterically Regulated Electronic Properties. *J. Am. Chem. Soc.* **2012**, *134*, 16921–16924.

5. Lübbecke, H.; Boldt, P. Quinones—VII. *Tetrahedron* **1978**, *34*, 1577–1579.

6. Fulmer, G. R.; Miller, A. J. M.; Sherden, N. H.; Gottlieb, H. E.; Nudelman, A.; Stoltz, B. M.; Bercaw, J. E.; Goldberg, K. I. NMR Chemical Shifts of Trace Impurities: Common Laboratory Solvents, Organics, and Gases in Deuterated Solvents Relevant to the Organometallic Chemist. *Organometallics* **2010**, *29*, 2176–2179.

7. Carey, M. C.; Adelman, S. L.; McCusker, J. K. Insights into the Excited State Dynamics of Fe(II) Polypyridyl Complexes from Variable-Temperature Ultrafast Spectroscopy. *Chem. Sci.* **2019**, *10*, 134–144.

8. Krishna, A.; Fritsch, L.; Steube, J.; Argüello Cordero, M. A.; Schoch, R.; Neuba, A.; Lochbrunner, S.; Bauer, M. Low Temperature Emissive Cyclometalated Cobalt(III) Complexes. *Inorg. Chem.* **2025**, *64*, 1401–1409.

9. Yarranton, J. T.; McCusker, J. K. Ligand-Field Spectroscopy of Co(III) Complexes and the Development of a Spectrochemical Series for Low-Spin d^6 Charge-Transfer Chromophores. *J. Am. Chem. Soc.* **2022**, *144*, 12488–12500.

10. Chan, A. Y.; Ghosh, A.; Yarranton, J. T.; Twilton, J.; Jin, J.; Arias-Rotondo, D. M.; Sakai, H. A.; McCusker, J. K.; MacMillan, D. W. C. Exploiting the Marcus Inverted Region for First-Row Transition Metal–Based Photoredox Catalysis. *Science* **2023**, *382*, 191–197.
